# Serum Metabolomics Analysis of Asthma in Different Inflammatory Phenotypes: A Cross-Sectional Study in Northeast China

**DOI:** 10.1155/2018/2860521

**Published:** 2018-09-23

**Authors:** Zhiqiang Pang, Guoqiang Wang, Cuizhu Wang, Weijie Zhang, Jinping Liu, Fang Wang

**Affiliations:** ^1^Department of Pathogen Biology, College of Basic Medical Sciences, Jilin University, Changchun, China; ^2^School of Pharmaceutical Sciences, Jilin University, Changchun, China; ^3^Third Department of Respiratory Disease, Jilin Provincial People's Hospital, Changchun, China

## Abstract

**Background and Objective:**

Asthma as a chronic heterogeneous disease seriously affects the quality of life. Incorrect identification for its clinical phenotypes lead to a huge waste of medical resources. Metabolomic technique as a novel approach to explore the pathogenesis of diseases have not been used to study asthma based on their clear defined inflammatory phenotypes. This study is aimed to distinguish the divergent metabolic profile in different asthma phenotypes and clarify the pathogenesis of them.

**Methods:**

Participants including eosinophilic asthmatics (EA, n=13), noneosinophilic asthmatics (NEA, n=16), and healthy controls (HC, n=15) were enrolled. A global profile of untargeted serum metabolomics was identified with Ultra Performance Liquid Chromatography–Mass Spectrometry technique.

**Results:**

Multivariate analysis was performed and showed a clear distinction between EA, NEA, and HC. A total of 18 different metabolites were recognized between the three groups based on OPLS-DA model and involved in 10 perturbed metabolic pathways. Glycerophospholipid metabolism, retinol metabolism, and sphingolipid metabolism were identified as the most significant changed three pathways (impact > 0.1 and -log(*P*) > 4) between the phenotypes.

**Conclusions:**

We showed that the different inflammatory phenotypes of asthma involve the immune regulation, energy, and nutrients metabolism. The clarified metabolic profile contributes to understanding the pathophysiology of asthma phenotypes and optimizing the therapeutic strategy against asthma heterogeneity.

## 1. Introduction

Asthma as a chronic life-threatening respiratory disease, which is driven by heterogeneous inflammatory mechanisms, has a 7–10% prevalence worldwide. Its morbidity is increasing with the change of environment [1, 2]. The pathogenesis of asthma is complicated and attributed to the interaction among genetic, epigenetic, and environmental factors [3]. Different asthmatics show a distinct, sometimes completely refractory response to the recommended standard treatment and, therefore, require a large amount of medical resource for management [4]. Asthma manifests clinically with two typical phenotypes as eosinophilic asthma (EA) and noneosinophilic asthma (NEA) [5], whose classification is based on the ratio of granulocytes in peripheral blood or sputum [6, 7]. Generally, EA usually represents more severe airway hyperresponsiveness and a higher risk of exacerbation [8]. As for NEA, mainly consisting of neutrophilic asthma shows a poorly controlled and a worse airway obstruction status [9, 10]. The underlying disease mechanism, as related to inflammatory phenotypes, is critical for clinical therapy and has been the subject of recent reports but remains poorly understood.

Given the complexity and heterogeneity of asthma, the advanced methods such as multiomics techniques are urgently needed to illustrate its pathogenesis. Metabolomics is a high-throughput technique for multitargeted analysis of endogenous metabolites (<1 kDa) generated by biochemical reactions under a given set of physiological conditions and has been widely used to identify novel biomarkers and understand the molecular mechanisms of diseases [3, 11]. As one of the systemic biology researching approach, it focused on what had happened and changed in vivo. This is different from other omics methods, such as genomics and transcriptome, which could only tell us what might happen. Metabolome has even been claimed as “the best indicator of an organism's phenotype” [12]. Metabolomics has been increasingly explored in multiple biological samples from asthmatics such as urine, plasma, and exhaled breath condensate [3, 13, 14] to achieve an improved understanding of asthma. Despite the researches in asthma metabolomic to date have been diverse, most studies describe asthma as a single disease inflammatory phenotype. In addition, the diversity of analyzing technology and sample types make it nearly impossible to identify the cause and mechanism hidden behind the two asthma inflammatory phenotypes.

How metabolites in asthmatics with different clinical inflammatory phenotypes changed and participated in the different inflammation process is rarely reported yet. Herein, we investigated the metabolic profile in asthmatics with different phenotypes and healthy controls by applying Ultra Performance Liquid Chromatography–High Resolution Mass Spectrometry (UPLC-MS/MS) techniques. The aim of this research is to determine the metabolic signatures and the related metabolic pathways. Therefore, we tested the hypothesis that there existed a characteristic metabolites difference profile between the two inflammatory asthma phenotypes and, moreover, the associated metabolic pathways played a potential role in elucidating the mechanism of asthma heterogeneity.

## 2. Methods and Materials

### 2.1. Subjects Recruitment and Ethic Assessment

All volunteers including asthmatics and healthy controls (HC) were recruited from the Northeast China Asthma Network Center (People's Hospital of Jilin Province and The Second Hospital of Jilin University). All asthmatics were diagnosed as mild to moderate asthma according to the Global Initiative for Asthma (GINA) guidelines (updated in 2016) [15], based on current respiratory symptoms and evidence from spirometry.

Inhaled corticosteroid (ICS) and long acting beta agonists were ceased for 24 hours; then spirometry before and after bronchodilator treatment with salbutamol was performed according to our standard protocol [16]. The reversibility of FEV1 to salbutamol was more than 12% and 250 mL. Current smokers, ex-smokers, and those with a recent respiratory tract infection were excluded. Patients with ACQ6 scores < 1.5 were included. Healthy volunteers were not diagnosed with any diseases or any history of chronic lung diseases.

This study was approved by the Jilin Province People's Hospital Ethics Committee and registered at the International Clinical Trials Registry Platform and Chinese Clinical Trial Registry (NO. ChiCTR-COC-16008287). Written informed consent was provided by all participants.

### 2.2. Sample Collection and Preparation

To avoid variation from circadian rhythms, fasting peripheral whole blood and serum were drawn in the morning between 8:00 and 10:30 AM as described previously [17, 18] for full blood count, total IgE quantification, and serum metabolomic study. Asthmatic patients were clustered into EA and NEA phenotypes according to a previously reported discriminant calculation formula based on the blood cell parameters [19]. Briefly, we calculate score1 and score2 as follows: (1)* calculate score 1 = -9.5243 + [70.0975 × eosinophil/lymphocyte] - [3.7790 × natural log (eosinophil/neutrophil)]*; (2)* calculate score = -14.5853 + [101.2198 × eosinophil/lymphocyte] - [3.9567 × natural log (eosinophil/neutrophil)]*. Then we use the following decision rule: if score1 > score2, cluster the subjects into NEA group or, otherwise, to EA group.

All serum samples were aliquoted and stored in -80°C for analysis and then thawed on ice. Additional methanol (600 *μ*L, methanol (HPLC), Fisher Chemical, Cat. A452-1) was added to the serum (200 *μ*L) for each sample and vortexed for 3 min. After settling at ice for 15 min, all samples were centrifuged at 12,000g for 10 min at 4°C so as to make samples free of protein. Then the supernatant (500 *μ*L) was collected and lyophilized at -60°C and 10.0 pa air pressure for 24 hours. The lyophilized residue was redissolved in 100 *μ*L of methanol-water (4 : 1, v/v); after centrifugation for 15 min at 12,000 g, an aliquot of 2 *μ*L was injected for UPLC-MS/MS analysis.

### 2.3. UPLC-MS/MS Procedure

The serum UPLC analysis was performed with Waters ACQUITY UPLC system (Waters Corporation, Milford, MA, USA), which had been equipped with a BEH C18 column (2.1 mm× 100 mm, 1.7 mm, Waters Corporation, Milford, MA, USA). The temperature of the UPLC column and autosampler was set as 30°C and 15°C, respectively. The flow rate was set as 0.4 mL/min. The mobile phase was composed of eluent A (0.1% formic acid in water, v/v) and eluent B (0.1% formic acid in acetonitrile, v/v). The gradient procedure was optimized as follows: 10% B from 0 to 2 min, 10–90% B from 2 to 26 min, 90% B from 26 to 28 min, 90–10% B from 28 to 28.1 min, and 10% B from 28.1 to 30 min. The different ratios of acetonitrile/water were mixed as strong wash solvent (90/10, v/v) and weak wash solvent (10/90, v/v).

A Waters Xevo G2-S Quadrupole Time-Of-Flight (QTOF) mass spectrometer (Waters Corporation, Milford, MA, USA) connected to the UPLC system was utilized to carry out the mass spectrometry with an electrospray ionization in both positive (ESI^+^) and negative (ESI^−^) ion modes. The instrumental parameters were optimized as follows: capillary voltages were 2.6 kV (ESI^+^) or 2.2 kV (ESI^−^), sample cone voltage was 40 V for both ESI^+^ and ESI^−^. In addition, source temperature was set as 120°C with cone gas flow rate at 50 L/h and desolvation temperature was at 300°C with desolvation gas rate flow at 800 L/h. Collision energy of low energy and ramp collision energy of high energy was set at 6.0 V and at 20–40 V, respectively. Leucine-enkephalin (m/z 556.2771 in ESI^+^; m/z 554.2615 in ESI^−^) was used as the lock-mass in all analyses at a concentration of 300 ng/mL and flow rate of 20 *μ*L/min. The scan time and internal delay were set as 0.15 s and 0.02 s. Data was collected over a range of m/z 100-1500 with the calibration by sodium formate.

To ensure the stability and suitability of MS analysis, a quality control (QC) sample was prepared by pooling the same volume (20 *μ*L) from every serum samples. The QC sample was prepared in the same way as the other samples above. Ten chromatographic peaks of ions with high abundances from the QC sequencing datasheet were selected to evaluate the validation of systematic method. The validation of the methodology was finished before the injection of all samples. The repeatability of the chromatogram and spectrum system was evaluated by analyzing 6 successive injections of the same QC sample before the work list in both ESI+ and ESI- modes, respectively. Intermediate precision on spectrum and chromatogram was also estimated by detecting 6 replicates of a serum sample in both ESI modes, respectively. Another 6 QC injections were performed randomly through the whole work list according to a previous report [20]. Full scan data of all samples was collected for further analysis.

### 2.4. Data Processing

The preprocessing of raw data produced by the mass spectrometer was finished with MarkerLynx XS V4.1 software for alignment, deconvolution, and data reduction so as to pair the mass and retention time with the corresponding intensities of all detected peaks. The main parameters were set similarly as before [21]. Briefly, retention time ranges from 0 to 29 min, mass ranges from 100 Da to 1,200 Da, mass tolerance is 0.10, minimum intensity is 5%, marker intensity threshold is 2000, mass window is 0.10 Da, retention time window is 0.20 min, and noise elimination level is 6. The processed files of ESI^+^ and ESI^−^ modes were exported for analysis.

### 2.5. Statistic and Bioinformatic Analysis

The exported data above were imported to SIMCA-P software (v14.1, Umetric, Umeå, Sweden) for carrying out multivariate analysis, including principle component analysis (PCA) and orthogonal projections to latent structures discriminant analysis (OPLS-DA), which was based on the known phenotyping clusters. The OPLS-DA was used to find potential biomarkers that significantly contributed to the metabolic distinction between the groups. In detail, the OPLS-DA models were established as EA versus NEA, EA versus HC, NEA versus HC, and asthmatics (joined EA and NEA) versus HC in both ESI^+^ and ESI^−^ modes, respectively. Variable importance of project (VIP) values was also estimated statistically. The relative standard deviation (RSD) also was calculated for the pooled QC injections to assess the quality and stability of MS data.

The difference in metabolites was identified by matching accurate mass to the Human Metabolome Database (HMDB Version 4.0) [22], with confirmation determined by comparing characteristic tandem mass spectrometry (MS/MS) fragmentation patterns. All metabolites included in statistical analyses were confirmed by MS/MS according to METLIN's high resolution tandem mass spectrometry (MS/MS) database [23] and HMDB database [22] with the parameters set as follows: adducts were M+H⌉^+^ and M+Na⌉^+^ for ESI^+^ and M-H⌉^−^ and M+FA⌉^−^ for ESI^−^. The tolerance of mass was set as 10 ppm. Some metabolites were further demonstrated by referring the chemical standards. The identification and comparison of some metabolites against the chemical standard samples were performed according to the retention time and the MS/MS fragments. After finishing the confirmation of the metabolites, all distinct metabolites were analyzed with MetaboAnalyst 4.0 for metabolomic pathway analysis [24]. The comprehensive metabolic network was constructed with Cytoscape Software (v3.6.1) [25] based on the data from Kyoto Encyclopedia of Genes and Genomes (KEGG,* updated in April 16, 2018*) database [26].

Statistical homogeneity of variance was estimated firstly by using the one-way ANOVA F-test and then the false discovery rate (FDR) test to avoid the false positive results. Kolmogorov-Smirnov test was used to ensure the normality of the data. Student's* t*-test for data with homogeneity of variance or Welch's* t*-test was performed for pairwise two-group analysis. Moreover, multiple comparisons among groups were performed by one-way analyses of variance (ANOVA) as described previously [27]. Mann-Whitney-Wilcoxon test was performed for the dataset, which does not follow the normality. All statistical significance was accepted at* P* < 0.05. The statistical analysis was completed with R (v. 3.3.3) basic statistical packages.

## 3. Results

### 3.1. Characteristics of All Participants

A total of 29 asthmatics were recruited and diagnosed. Asthmatics who met the inclusion criteria were included and classified as EA (n=13) and NEA (n=16) phenotypes. 15 healthy aged-matched volunteers were also included. Clinical demographics of the study cohort are presented in Table 1. The statistics of basic natural characteristics, including gender, age, and BMI values, do not display any significant difference between the three groups. The serum total IgE level and blood eosinophil ratio are significantly higher in EA than in the other groups. But the neutrophil ratio in EA was lower than NEA. Both EA and NEA showed reduced spirometry and FEV1/FVC ratio.

BMI is body mass index; ACQ6 is asthma control questionnaire 6; FEV1 is forced expiratory volume in one second; FVC is forced vital capacity; Score1 is one of the calculating results of clustering formula; Score2 is the other calculating result. If Score1 is less than Socre2, clustering the sample as eosinophilic asthma or otherwise as noneosinophilic asthma.

### 3.2. Multivariate Analysis of Metabolomic Data

**Figure 1:**
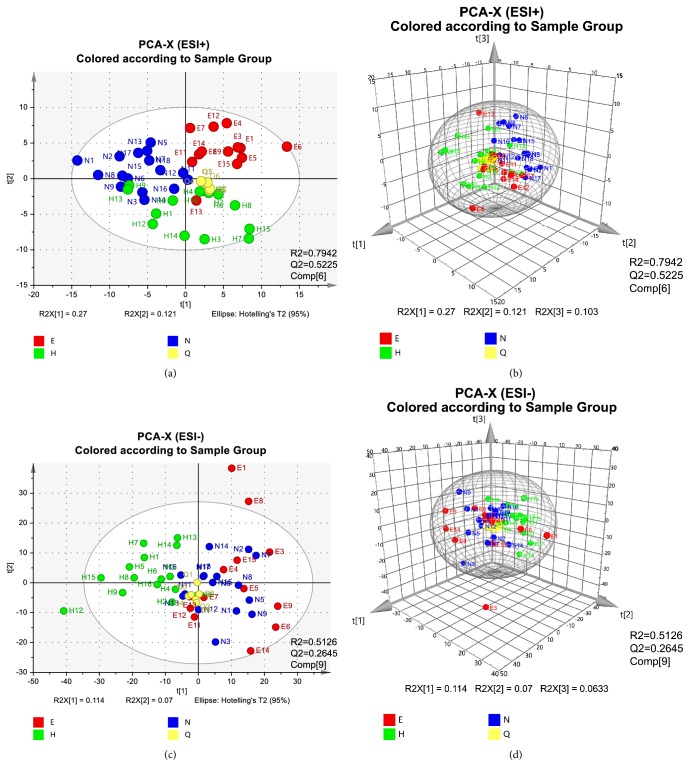
PCA analysis results of UPLC-MS. All samples in ESI^+^ modes distributed in 2-dimensional plot (a) and in 3-dimensional plot (b). All samples in ESI^−^ modes distributed in 2-dimensional plot (c) and in 3-dimensional plot (d). To avoid a too complicated plot, EA was abbreviated as E, NEA as N, HC as H, and QC as Q (PC1=27.5%, PC2=12.6%, and PC3=10.7% for ESI^+^ and PC1=11.6%, PC2=7.08%, and PC3=6.43% for ESI^−^).

All RSD values of spectrum and chromatogram, including repeatability and intermediate precision, were calculated as less than 3.5 %, which met the requirement for the subsequent analysis according to a previous report [28] (Table S1). Then multivariate analysis was performed with SIMCA-P software. PCA as an unsupervised lowering-dimension pattern recognition model was firstly established based on the spectra of samples to discern the presence of inherent similarities in mass spectral profiles as displayed in Figure 1. The separated groups showed intrinsic variation among all groups, especially in ESI^+^ ion mode (R2=79.42%, Q2=52.25%). Total of 4200 features were obtained from 4214 features in raw data to construct the PCA and OPLS-DA models in ESI^+^. At least 1594 features were also obtained from 1621 features in raw data to construct the PCA or OPLS-DA models in ESI^−^. Besides, the QC injections were clustered tightly in PCA indicating a satisfactory stability of the system.

**Figure 2:**
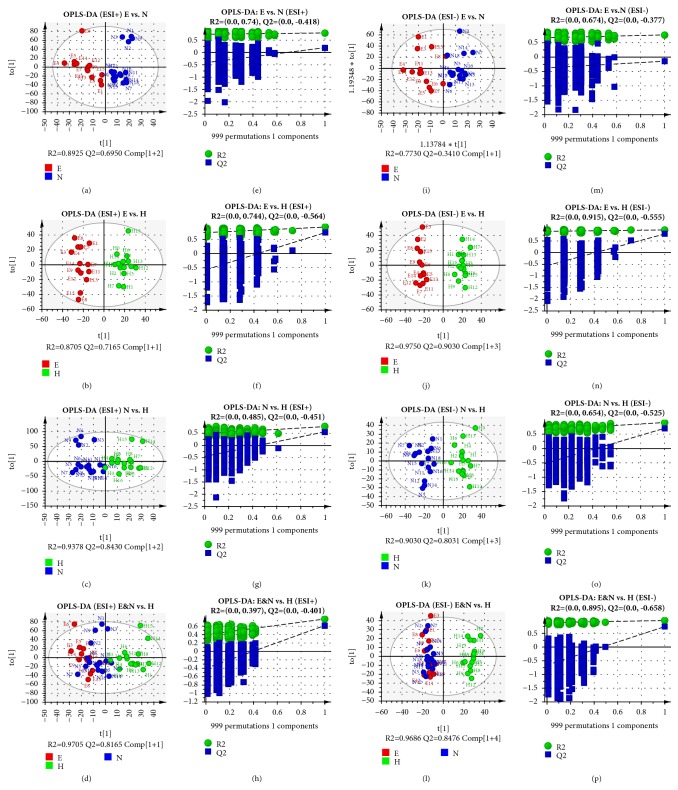
OPLS-DA score plots and permutation tests results. The OPLS-DA discrimination and permutation tests between different groups: (a & e) EA versus NEA in ESI^+^,* P* = 0.0012; (b & f) EA versus HC in ESI^+^,* P* < 0.0001; (c & g) NEA versus HC in ESI^+^,* P* = 0.0002; (d & h) Asthma versus HC in ESI^+^,* P* < 0.0001; (i & m) EA versus NEA in ESI^−^,* P* > 0.05; (j & n) EA versus HC in ESI^−^,* P* < 0.0001; (k & o) NEA versus HC in ESI^−^,* P* < 0.0001; (l & p) Asthma versus HC in ESI^−^,* P* < 0.0001. EA was abbreviated as E, NEA as N, and HC as H. All* P* values were estimated with CV-ANOVA test. Ellipse in the OPLS-DA panels represents Hotelling's T2 test with the confidence interval of 0.95.

A total of 8 OPLS−DA models were constructed based on the PCA results to discriminate the difference under the already established separation between different groups. As shown in Figure 2, the subjects in different groups were appreciably separated from each other in both ESI^+^ and ESI^−^ modes indicating that there exists no extremely abnormal sample. In addition, OPLS-DA, which had been used to maximize the covariance of the measured data, was validated with permutation tests (n=999) [29–32]. After the sufficient permutation test, the lines of grouping samples were significantly located underneath the random sampling lines (Q2 < 0.05 for all models [33]), which indicated a fine validity for the following characteristic metabolites biomarkers identification. The validation of all OPLS-DA models was also performed with leave-1/7-out method as described previously [34–37]. R2X, R2Y, and Q2 values of the cross-validation have been provided in Table S3. Q2 values, which reflected the predictability of the models exceeded 0.5 except E versus N in ESI^−^ model (Figure 2(m)).

### 3.3. Global Profiles of Distinct Metabolites

The potential differential metabolites were chosen according to the contribution of Variable Importance for the Projection (VIP) that were extracted from the OPLS-DA models above. Metabolites were selected when the VIP values exceed 1.0 and* P* values, calculated statistically with t-test or Mann-Whitney-Wilcoxon test being less than 0.05 [27]. Based on these criteria, a total of 18 remarkably changed metabolites in sera were determined with the details in Table 2.

**Figure 3:**
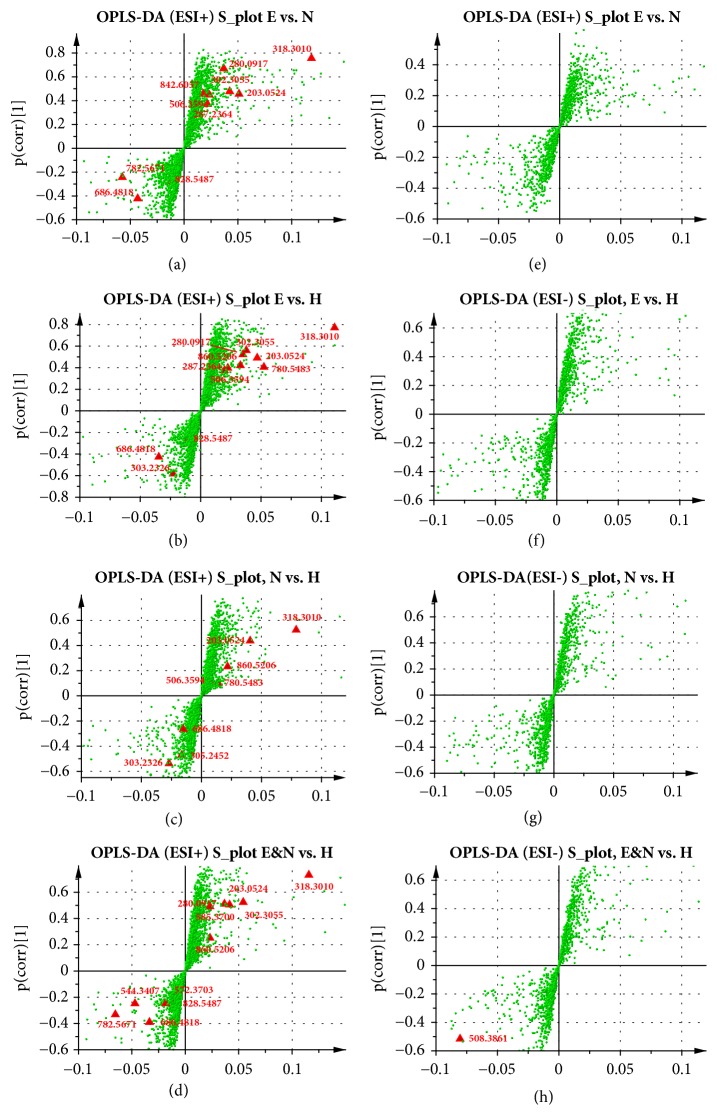
S-plots of all OPLS-DA models. All identified metabolites in different OPLS-DA model, including EA versus NEA in ESI^+^ (a) and in ESI^−^ (e); EA versus HC in ESI^+^ (b) and in ESI^−^ (f); NEA versus HC in ESI^+^ (c) and in ESI^−^ (g); Asthma versus HC in ESI^+^ (d) and in ESI^−^ (h). All significantly changed metabolites identified were marked as red triangle. Other components were green points. The detailed information on the correlation and covariation values about every distinct metabolites were summarized in Table S4.

Identification of the metabolites was performed based on the accurate mass and displayed in S-plots as Figure 3 for potential biomarkers. The S-plots were marked based on the metabolic profiles between all groups and indicated that 18 ions contributed to the clustering, with retention time and m/z pairs as follows: 0.61_280.0917** (S1)**, 0.70_203.0524** (S2)**, 5.91_860.5206** (S3)**, 5.99_585.37** (S4)**, 6.89_842.6057** (S5)**, 11.06_828.5487** (S6)**, 12.90_318.301** (S7)**, 15.19_302.3055** (S8)**, 16.65_544.3407** (S9)**, 18.13_303.2326** (S10)**, 18.81_572.3703** (S11)**, 19.18_506.3594** (S12)**, 22.81_305.2452** (S14)**, 25.80_287.2364** (S15)**, 26.79_686.4818** (S16)**, 27.99_782.5671** (S17)**, and 28.41_780.5483** (S18)** in ESI^+^ mode, and 20.06_508.3861** (S13)** in ESI^−^ mode. The detailed information of fragmentation used for the identification of all metabolites was included in Table 2. The original MS/MS spectra and the referenced spectra from HMDB or METLIN database of every metabolites were provided in Figures S1–S18.

The monosaccharides identified according to the database Figures S2-2, including potential Myoinositol, D-mannose, Beta-d-glucose, and D-tagatose, and retinols (Figure S15) including potential 9-cis-retinol, 11-cis-retinol, and All-trans-retinol were further identified based on their standard chemicals. The MS/MS spectra of all standard chemicals were provided in Figures S2 and S15. LysoPCs including LysoPC(18:1), LysoPC(p-18:1), and LysoPC(o-18:0) were further identified based on their fragments of heads (Figures S9, S12, and S13) and tails in MS/MS. In detail, the positive ions with m/z 240.0995 are C8H19NO5P⌉^+^, which is the head of LysoPCs being observed in all LysoPCs including S9, S12, and S13. The tail of LysoPC(18:1) and the negative ion C18H31O⌉^−^ with m/z 263.2308 were observed in MS/MS spectra of** S9**. The tail of LysoPC(p-18:1) and the negative ion C18H33O⌉^−^ with m/z 265.2576 were observed in MS/MS spectra of** S12**. The tail of LysoPC(o-18:0) and the negative ion C18H37O⌉^−^ with m/z 269.2875 were observed in MS/MS spectra of** S13**.

**Figure 4:**
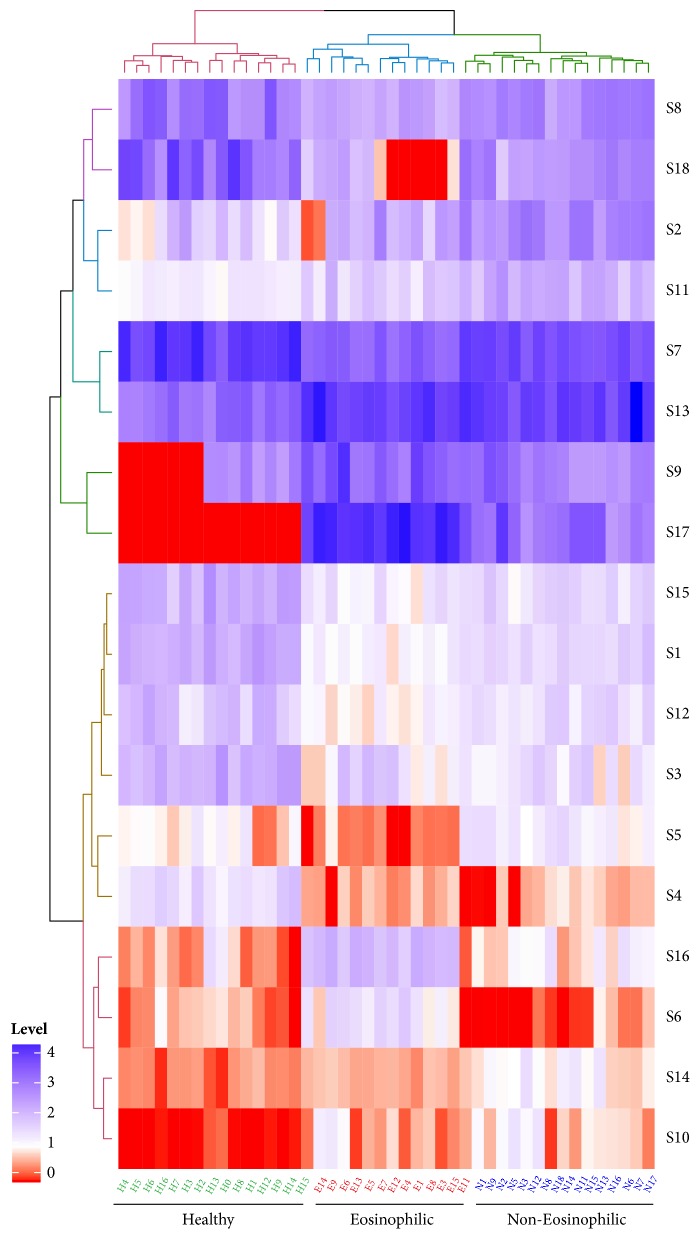
Heatmap of all distinct metabolites.

All distinct metabolites identified in present study were displayed straightforward with a heatmap in Figure 4. All serum samples were clustered as three groups, which were consistent with the asthmatic phenotyping or healthy conditions. Besides, the content levels of all differentiated metabolites including the qualitative comparison, fold change, and the confidence intervals were summarized in Table 3.

### 3.4. Metabolic Pathways

**Figure 5:**
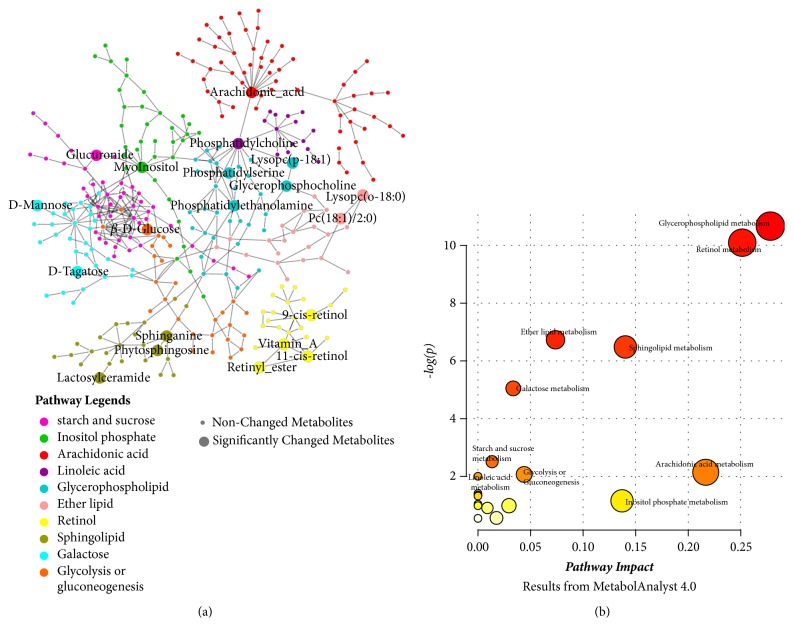
The perturbed metabolic network of the potential biomarker associated allergic asthma and the pathway impact extent according to the MetaboAnalyst 4.0 results. (a) The network of all metabolic pathways related to the pathogenesis of asthma including the significantly changed network, potential related, and trend-changing ones. (b) Summary of all pathway statistical analysis from MetaboAnalyst 4.0.

The most relevant metabolic pathways related to the inflammatory phenotypes of asthma was systematically investigated and identified. 10 metabolic pathways were discovered to be related to the pathogenesis of asthma: glycerophospholipid metabolism** (M1)**, retinol metabolism** (M2)**, sphingolipid metabolism** (M3)**, ether lipid metabolism** (M4)**, galactose metabolism** (M5)**, arachidonic acid metabolism** (M6)**, inositol phosphate metabolism** (M7)**, starch and sucrose metabolism** (M8)**, linoleic acid metabolism** (M9)**, and glycolysis or gluconeogenesis metabolism** (M10)**. The interaction network was constructed according to the KEGG database and shown in Figure 5(a).** M2** seems to be separated with other pathways in the network.

The perturbed pathways related metabolism in sera had been summarized and reported in Table 2. Despite a total of 10 metabolic pathways were recognized, different pathways were affected in different extent according to their impact values and* P* values (Table S2). As shown in Figure 5(b), 3 metabolic pathways displayed significant changes (impact > 0.1 and -log(*P*) > 4) including** M1-M3**; 4 metabolic pathways showed a potential relationship with the variation of pathways (impact > 0.1 or -log(*P*) >2) including** M4-M7**. The other pathways just show a changing trend (-log(*P*) >2) including** M8-M10**.

Besides, the changed pathways showed a discrepancy in different OPLS-DA comparison models. Briefly,** M1-M3**,** M5,** and** M7** were changed between EA and NEA.** M1-M3**,** M5**,** M7,** and** M9** were changed between EA and HC.** M1, M3, **and** M5-M9** were changed between NEA and HC.** M1-M5** and** M7-M10** were changed between asthma and HC. The detailed statistical changes of every metabolites involved in different groups were also summarized in Table 3.

## 4. Discussion

In the present study, we have reported the distinct metabolic profile between the different clinical inflammatory phenotypes and healthy subjects in Northeast China. All differential metabolites in sera were identified with UPLC-MS/MS techniques. Multivariate analysis was performed to clarify the difference within all groups. Eight OPLS-DA models were established and 7 of them displayed a reliable predictive effect. As a result, we characterized 18 distinct metabolites and 10 perturbed metabolic pathways based on the models with robust reliability. Glycerophospholipid, retinol, and sphingolipid metabolism, the top 3 significantly changed metabolic pathways, have been determined. The changed metabolic pathways varied across the different multivariate models indicating an unestablished mechanism.

A large quantity of researches on asthma metabolomics have been performed extensively until now. Current studies have mainly focused on the difference between asthmatics and healthy subjects or on distinct asthma severity [3, 38]. Many biomarkers have been discovered, such as saturated fatty acids and ammonium ion [39]. All related studies had already been reviewed recently [2]. As for the metabolome research on inflammatory phenotype, no association between metabolic profile with sputum eosinophilia was reported before [3]. However, Loureiro et al. studied the urinary metabolomics and demonstrated that lipidic peroxidation was related to the clinical characteristics of nonobese asthmatics, such as eosinophilic inflammation [13]. Ibrahim et al. tried to classify asthma phenotypes defined by many clinical factors including sputum cell profiles with metabolomic techniques but only identified the difference based on the ICS and sputum neutrophilia [14]. In this study, the metabolic profile variation between different clinical inflammatory phenotypes was concerned about for the first time. How these differential pathways identified play their roles in the pathogenesis of asthmatic phenotypes deserves the following attention.

Glycerophospholipids are a critical series compounds for constituting the cell membrane structure and participating in many biological regulatory processes, including the pathogenesis of asthma [40]. As the most important ones, phosphatidylcholines (PCs) nearly make up approximately half of the total cellular phospholipids [41] and correlated with eosinophilic cationic protein in sputum [42]. PCs showed a higher level in asthmatics in serum but a decrease in lungs [42, 43]. In this study, we identified 2 homologues of PCs, which showed the contrary comparison result between the two phenotypes and a converse trend compared with healthy controls. Different from PCs, lysophosphatidylcholines (LysoPCs) have been reported to display a reduced level in experiment asthmatic mice and may act as immune suppressors [44]. In addition, phosphatidylserine (PS) was once considered to play an important role in Th2 induction and airway hyperreactivity [45], despite its regulatory effect on mast cells being converse to the above [46]. The distinct changing pattern for PCs, LysoPCs, and PSs indicates that there exist different roles of phospholipids homologues in the pathogenesis of asthma.** S16** (PE) was higher in asthmatics, especially in EA. Moreover, glycerophosphocholine in a lower level of for asthmatics may be related to its anti-inflammatory effect [47].

It is noteworthy that the production of LysoPCs from the hydrolysis of phospholipids is always accompanied with the production of arachidonic acid, who is a precursor molecule for various proinflammatory eicosanoids [48]. Our results also imply a potential differential role of arachidonic acid metabolism related immune mechanism in NEA, although different arachidonic acid metabolites may have the distinct pro- or anti-inflammatory effect. Serum plasmalogens (ether lipid) as a critical component associated with oxidative stress and chronic inflammation were also discovered to be related to the pathogenesis of asthma for the first time but not the inflammatory phenotypes. In line with the previous studies, we observed a potential higher activation of linoleic acid metabolism, which had been reported to be tightly associated with the production of arachidonic acid [49] although not any significant distinction was observed between the phenotypes.

The change of energy metabolism and trigger of airway inflammation had already been investigated extensively. In this study, we also identified a variety of hexoses, which were generally higher in asthmatics, especially in NEA. The higher carbohydrates, such as glucose, have the potential to induce the generation of reactive oxygen species, a second signal for inflammasome activation [50]. Glucuronide was identified specifically to be related to the change of starch and sucrose metabolism. Besides, glycolysis or gluconeogenesis pathway also showed a potential changing trend in asthmatic energy metabolic network. Inositol phosphate metabolism, which was associated with another differentiated hexose, myo-inositol, has been reported to participate in the regulation of the airway smooth muscle contractility [51].

Retinol has been reported to be involved in many physiological activities, such as embryonic development, cell growth and differentiation, and immune responses [52]. As an antioxidant, it plays a vital role in repair of the airway epithelium and the formation of lung primordium [53]. The level of retinol and its bioactive metabolite had been demonstrated; retinoic acid were lower in asthmatics, which might adversely affect lung development and promote AHR [54]. The deficiency of retinol shows the potential to induce and aggravate the existing inflammation via the activation of NF-*κ*B [55]. Consistent with the previous reports, the retinol level decreased in asthmatics in the present study, especially in EA, which may be related to the failure of suppressing the differentiation of eosinophil [56]. However, although the reduced level of retinol metabolism was observed in NEA, a significant difference from HC was not reported, which indicated a weaker impact of retinol deficiency on the pathogenesis of NEA than on EA.

More importantly, sphingolipid metabolism pathway was significantly perturbed in all OPLS-DA models. It is not surprising because the role of sphingolipids in the pathogenesis of asthma had already been studied extensively. Actually, sphingolipids as the highly bioactive compounds are involved in inflammation, airway smooth muscle contraction, etc. [20]. A critical asthma related protein, ORMDL3, has been reported to inhibit the activity of serine palmitoyl-transferase (SPT), the rate-limiting enzyme that catalyzes the first step of de novo biosynthesis of sphingolipids [57]. Consistent with the previous report [20], we also observed a decrease of phytosphingosine (S12) and sphinganine (S13) in asthmatics. The different decreasing degree of them in EA and NEA might be caused by the SNPs of ORMDL proteins in the asthmatics with different phenotypes [58]. Because of the comprehensive role of sphingolipids in vivo, the proinflammatory effect of some sphingolipids, such as sphingosine-1-phosphate, whose precursor is sphinganine cannot be identified in the present study. Besides lactosylceramide as a central precursor in the synthesis of gangliosides, sulfatides were identified as dysregulated. The distinct level of lactosylceramide in phenotypes indicated an unknown mechanism.

Many systematical biological studies on the pathogenesis of asthma had been performed, but the diversity of researching samples and techniques confined the complete comprehension of asthma heterogeneity. Mass spectrometry, as the most sensitive metabolomic technology, was utilized to detect all compounds and provided us with a large amount of metabolite data [59]. Despite that, the limited throughput seems to have a negative influence on the accuracy. Detection on serum presents a real-time global metabolites profile and has the potential to reveal the mechanism of the diseases. Compared with targeted metabolomic techniques, untargeted techniques could analyze all the measurable molecules in a sample including chemical unknowns. After the multivariate analysis, the data led to the identification of novel 24 biomarkers. However, there still existed a significant limitation due to the small sample size. In addition, we cannot identify the level of every homologues precisely, such as the hexoses in 0.70 retention minute for the extremely limited ability to quantify with the untargeted metabolomics. Future exploration with GC-MS techniques might be more appropriate to explore the roles of hexoses [60]. Nevertheless, this pilot study will be a pioneer for further determination on the discovered metabolism pathways with targeted metabolomic technique.

## 5. Conclusions

The present metabolomic research showed 5 perturbed metabolic pathways between the typical inflammatory phenotypes and 18 metabolites for potential diagnosis biomarker. These metabolic pathways identified involve the immune regulation, energy, and nutrients metabolism. The clarified metabolic profile contributes to understand the pathophysiology of asthma phenotypes but requires further targeted metabolomics to improve the therapeutic strategy.

## Figures and Tables

**Table 1:** Characteristics of all subjects.

	**Eosinophilic Asthma (EA)**	**Non-eosinophilic Asthma (NEA)**	**Healthy Control (HC)**
**Subjects Number**	13	16	15
**Gender (female/male)**	5/8	9/7	6/9
**Age/years** ^†^	38.38 (33.00, 44.00)	40.75 (35.25, 47.5)	38.47 (31.5, 44.5)
**BMI/(kg·m** ^**-2**^ **)** ^†^	24.09 (21.06, 26.21)	25.53 (21.56, 28.60)	22.99 (20.72, 25.81)
**Serum IgE/(ng·mL** ^**-1**^ **)** ^†^	212.7 (134.9, 275.8)^∗∗#^	102.6 (55.77, 132.1)	106.7 (76.00, 121.9)
**ACQ6** ^†§^	0.9677 (0.67, 1.00)	1.074 (0.67, 1.35)	NA
**FEV1/FVC (**%**)** ^†^	61.0 (54.0, 68.0)^∗∗^	63.6 (56.8, 70.3)^∗∗^	85.3 (82.5, 88.5)
**FEV1/FVC (**% **post)** ^†^	77.8 (74.0, 81.0)	78.5 (74.3, 83.8)	NA
**FEV1 Reversibility (mL)** ^†^	333.8 (310.0, 380.0)	330.3 (285.0, 362.5)	NA
**FEV1 Reversibility (**%**)** ^†^	16.8 (14.0, 19.0)	14.9 (12.8, 16.3)	NA
**Eosinophil (**%**)** ^†^	8.5 (6.0, 10.0) ^∗∗##^	1.3 (1.0, 2.0)	2.0 (1.0, 2.3)
**Neutrophil (**%**)** ^†^	56.2 (50.0, 59.0)^#^	64.8 (59.3, 70.3)	59.75 (56.5, 64.0)
**Score1** ^†^	20.46 (10.95, 19.82)^#^	9.47 (8.23, 10.25)	NA
**Score2** ^†^	25.70 (11.71, 25.71)^#^	6.37 (4.54, 6.26)	NA

^†^Data are shown as mean (Q1, Q3). ^§^ACQ6 is the average of ACQ6 scores according to the GINA guidelines *(updated in 2016*)[15]. ^∗^*P* < 0.05 versus healthy control group. ^∗∗^*P *< 0.01 versus healthy control group. ^#^*P* < 0.05 versus noneosinophilic asthma group. ^##^*P* < 0.01 versus noneosinophilic asthma group.

**Table 2:** Identification results of all metabolite compounds in the serum of all subjects.

**NO**	**tR/min**	**Mass/Da**	**E** **S** **I** ^†^	**VIP**	**Formula**	**Compound**	**KEGG ID**	**MS** ^**E**^ **F** **r** **a** **g** **m** **e** **n** **t** **a** **t** **i** **o** **n** ^§^	**Error/ppm**	**Pathway**
**S1**	0.61	280.0917	+	2.51	C_8_H_20_NO_6_P	Glycerophosphocholine	C00670	80.9704, **104.1085**, 166.0630, 256.1188	1	Ether lipid & Glycerophospholipid
**S2** ^∗^	0.70	203.0524	+	2.82	C_6_H_12_O_6_	Monosaccharides	C00137	85.0292, 103.0079,** 135.0020**, 145.0090, 150.9735, 180.9964, 203.0503	1	Galactose, Inositol phosphate metabolism, Starch and sucrose & Glycolysis or Gluconeogenesis
**S3**	5.91	860.5206	+	2.08	C_46_H_80_NO_10_P	PS(18:0/22:5)	C02737	88.0626, 267.2686, 325.6945, 751.4440, **821.5210**	1	Glycerophospholipid
**S4**	5.99	585.37	+	1.74	C_33_H_54_O_7_	Cholesterol glucuronide	C03033	**85.0627**, 177.1049, 261.1591, 545.3312	10	Starch and sucrose
**S5**	6.89	842.6057	+	1.99	C_44_H_86_NO_10_P	PS(18:0/20:0)	C02737	86.0997, **249.1219**, 313.1090, 462.2505, 803.7540	3	Glycerophospholipid
**S6**	11.06	828.5487	+	1.19	C_42_H_79_NO_13_	Lactosylceramide (d18:1/12:0)	C01290	85.0646, 209.1875, **626.0863**, 788.5036	3	Sphingolipid
**S7**	12.90	318.301	+	7.38	C_18_H_39_NO_3_	Phytosphingosine	C12144	71.0698, 141.1184, 300.2883, **318.3008**	2	Sphingolipid
**S8**	15.19	302.3055	+	1.97	C_18_H_39_NO_2_	Sphinganine	C00836	69.0691, 137.0778, 187.1063, 284.2958, **302.3056**	0	Sphingolipid
**S9**	16.65	544.3407	+	3.05	C_26_H_52_NO_7_P	LysoPC(18:1)	C04230	86.0990, 104.1086, 184.0737, **240.0987**, 339.2748, 522.3394	2	Glycerophospholipid
**S10**	18.13	303.2326	+	1.7	C_20_H_30_O_2_	Retinyl ester	C02075	105.0726, **124.9997**, 177.1134, 191.1274	2	Retinol
**S11**	18.81	572.3703	+	1.2	C_28_H_56_NO_7_P	PC(18:1/2:0)	C04598	86.1000, **153.1025**, 184.0749, 235.1490, 479.3224	1	Ether lipid
**S12**	19.18	506.3594	+	1.07	C_26_H_52_NO_6_P	LysoPC(p-18:1)	C04230	86.0996, 125.0010, 166.0626, 184.0737, 240.1007, 249.1681, 309.3069, **506.3631**	2	Glycerophospholipid
**S13**	20.06	508.3861	-	3.2	C_26_H_56_NO_6_P	LysoPC(o-18:0)	C04317	152.9793, 168.2547, 240.0734, 283.4419, 421.3464, **508.3806**	3	Ether lipid
**S14**	22.81	305.2452	+	1.19	C_20_H_32_O_2_	Arachidonic acid	C00219	67.0587, 81.0723, 103.0763, 133.0868, 149.0226, **177.1091**, 185.0739, 191.1209, 235.1497	0	Arachidonic acid
**S15** ^∗^	25.80	287.2364	+	1.54	C_20_H_30_O	Retinols	C00473	79.0569, 105.0711, 121.1011, **133.0850**, 149.0295, 185.0760	2	Retinol
**S16**	26.79	686.4818	+	2.62	C_37_H_68_NO_8_P	PE(18:3/14:0)	C00350	183.2750, 261.1780, 476.3340, **686.4854**	9	Glycerophospholipid
**S17**	27.99	782.5671	+	3.39	C_42_H_82_NO_8_P	PC(16:0/18:1)	C00157	86.0991, 184.0737, 283.0508, 313.2929, **701.5731**	0	Arachidonic acid, Glycerophospholipid & Linoleic acid
**S18**	28.41	780.5483	+	3.08	C_44_H_78_NO_8_P	PC(20:4/16:1)	C00157	86.0996, 184.0748, 285.2657, 345.0710, 597.4855, **762.5735**	4	Arachidonic acid, Glycerophospholipid & Linoleic acid

^†^ESI^+^ (+): positive mode of electrospray ionization; ESI^−^ (-): negative mode of electrospray ionization. ^∗^Identified with reference standards. ^§^Tandem mass spectrum ion mass fragments in MS/MS. The characteristic fragments are highlighted as bold font. The unit of m/z is Th (Thomson).

**Table 3:** Statistic content level and changed fold of all metabolites.

**Metabolites**	**Levels**	**Changed Fold (EA/NEA/HC)**	***P* values** ^†^
S8	H>N>E	0.1019/0.3795/1.0000	0.0222
S18	H>N>E	0.0026/0.0517/1.0000	0.00023
S2	N>E>H	0.0170/0.1760/1.0000	0.00724
S11	N>E>H	1.2935/3.8047/1.0000	0.0048
S7	H>N>E	0.0000/0.0035/1.0000	<0.0001
S13	E≈N>H	51,928.0486/31,910.8200/1.0000	0.0016
S9	E>N>H	11,124.7683/10.9726/1.0000	0.000107
S17	E>N>H	3,159.1603/134.4344/1.0000	<0.0001
S15	H>N>E	0.3234/0.6369/1.0000	<0.0001
S1	H>N>E	0.1270/0.3526/1.0000	0.00327
S12	H>N>E	0.4664/0.9082/1.0000	0.000127
S3	H>N≈E	0.1537/0.1792/1.0000	<0.0001
S5	N≈H>E	0.8546/1.1421/1.0000	<0.0001
S4	H>N≈E	0.4628/0.4706/1.0000	0.00213
S16	E>N>H	12.7569/1.7083/1.0000	0.0156
S6	E>H≈N	1.4622/0.9640/1.0000	<0.0001
S14	N>E>H	1.1696/1.4421/1.0000	0.0086
S10	N≈E>H	2.2342/2.2794/1.0000	0.00802

^†^All  *P* values were calculated statistically by using one-way ANOVA for multiple comparison among all groups. ^§^The changed fold values of all distinct metabolites were the ratios of the exponential values of the average metabolites intensity in the group.

## Data Availability

The metabolomics raw data used to support the findings of this study are available from the corresponding author upon request.

## Supplementary Materials

Supplementary Materials Supplementary Table S1 described the details on the validation of systematic stability and suitability of UPLC-MS/MS. Supplementary Table S2 showed the metabolic pathways perturbed results from MetaboAnalyst 4.0. Figures S1 to S18 were the detailed mass spectra of all 18 metabolites and the results of the related database of METLIN or HMDB. Table S3 showed the validation results of all OPLS-DA models with leave-1/7-out approach. Table S4 listed the covariation values, correction values, VIP values, and P values of all metabolites in different OPLS-DA models.
