# Supplementary material for: Serum Metabolomics Analysis of Asthma in Different Inflammatory Phenotypes: A Cross-Sectional Study in Northeast China
Source: Biomed Res Int. 2018 Sep 23;2018:2860521. doi: 10.1155/2018/2860521 (PMC6174811; doi:10.1155/2018/2860521)
Supplement: Supplementary Materials — Supplementary Table S1 described the details on the validation of systematic stability and suitability of UPLC-MS/MS. Supplementary Table S2 showed the metabolic pathways perturbed results from MetaboAnalyst 4.0. Figures S1 to S18 were the detailed mass spectra of all 18 metabolites and the results of the related database of METLIN or HMDB. Table S3 showed the validation results of all OPLS-DA models with leave-1/7-out approach. Table S4 listed the covariation values, correction values, VIP values, and P values of all metabolites in different OPLS-DA models. [file 2860521.f1.pdf]

SUPPLEMENTARY MATERIALS

Table S1 Validation of systematic stability and suitability of UPLC-MS/MS

Modes	NO	RT/min	m/z	m/z in SPECTRUM		RT of CHROMATOGRAM		PA of CHROMATOGRAM	
				Repeatability (RSD%)	Precision (RSD%)	Repeatability (RSD%)	Precision (RSD%)	Repeatability (RSD%)	Precision (RSD%)
ESI^+	1	12.76	274.2747	0.0006	0.0008	0.0000	0.0000	0.1575	0.2530
	2	27.24	338.3433	0.0007	0.0019	0.0000	0.0000	0.5671	0.7452
	3	27.01	413.2671	0.0008	0.0012	0.0000	0.0000	0.7963	0.8931
	4	17.50	496.3404	0.0005	0.0013	1.4358	0.0000	0.1079	1.0480
	5	17.84	546.3561	0.0007	0.0015	0.0000	0.0000	0.1458	1.0021
	6	6.39	697.9091	0.0007	0.0009	2.4378	0.2033	2.4668	0.4056
	7	26.99	803.5423	0.0010	0.0004	0.3564	0.3801	2.7654	1.1395
	8	7.00	872.8603	0.0021	0.0000	0.5601	0.4385	1.1267	1.2263
	9	17.97	991.6704	0.0009	0.0021	1.1195	0.0410	0.1724	0.8215
	10	17.00	1039.6715	0.0007	0.0003	0.0000	0.0021	0.2348	0.4357
ESI^-	1	17.98	255.4359	0.0031	0.0012	0.0000	0.0000	0.9879	1.2931
	2	23.18	279.4293	0.0027	0.0011	0.0000	0.0031	0.1632	0.4155
	3	10.79	369.3278	0.0031	0.0004	0.0000	0.0000	0.1608	0.2078
	4	12.80	448.4175	0.0021	0.0019	0.0000	0.6581	0.0495	0.9550
	5	17.01	504.3253	0.0018	0.0000	1.0472	0.0504	0.1814	0.2165
	6	26.98	593.4810	0.0035	0.0010	0.2514	0.4162	2.6998	1.7362
	7	28.32	719.4469	0.0029	0.0024	0.4952	0.0000	3.4915	2.4671
	8	27.36	826.3910	0.0029	0.0002	1.1704	0.0000	2.1372	2.6638
	9	6.03	993.2782	0.0026	0.0014	1.9851	0.0042	1.3664	1.4521
	10	18.01	1035.2944	0.0032	0.0009	1.5668	0.0000	1.0324	2.4119

m/z, mass to charge ratio, Th; RT, retention time, min; PA, peak area. Precision is intermediate precision, RSD, Relative standard deviation.

Table S2 Metabolic pathways perturbed results from MetaboAnalyst 4.0.

Pathways	Total	Expected	Hits	Raw p	LOG(p)	FDR	Impact
Glycerophospholipid metabolism	39	0.34026	5	1.45E-05	11.145	0.001133	0.2782
Retinol metabolism	22	0.19194	4	2.83E-05	10.472	0.001133	0.25141
Ether lipid metabolism	23	0.20066	3	0.000907	7.0057	0.023286	0.07375
Sphingolipid metabolism	25	0.21811	3	0.001164	6.7556	0.023286	0.1402
Galactose metabolism	41	0.35771	3	0.004932	5.312	0.078913	0.03353
Starch and sucrose metabolism	50	0.43623	2	0.069103	2.6721	0.92138	0.01344
Arachidonic acid metabolism	62	0.54092	2	0.10026	2.3	1	0.21669
Glycolysis or Gluconeogenesis	14	0.12214	1	0.11575	2.1563	1	0.0439
Linoleic acid metabolism	15	0.13087	1	0.12351	2.0914	1	0
Inositol phosphate metabolism	39	0.34026	1	0.29142	1.233	1	0.13703

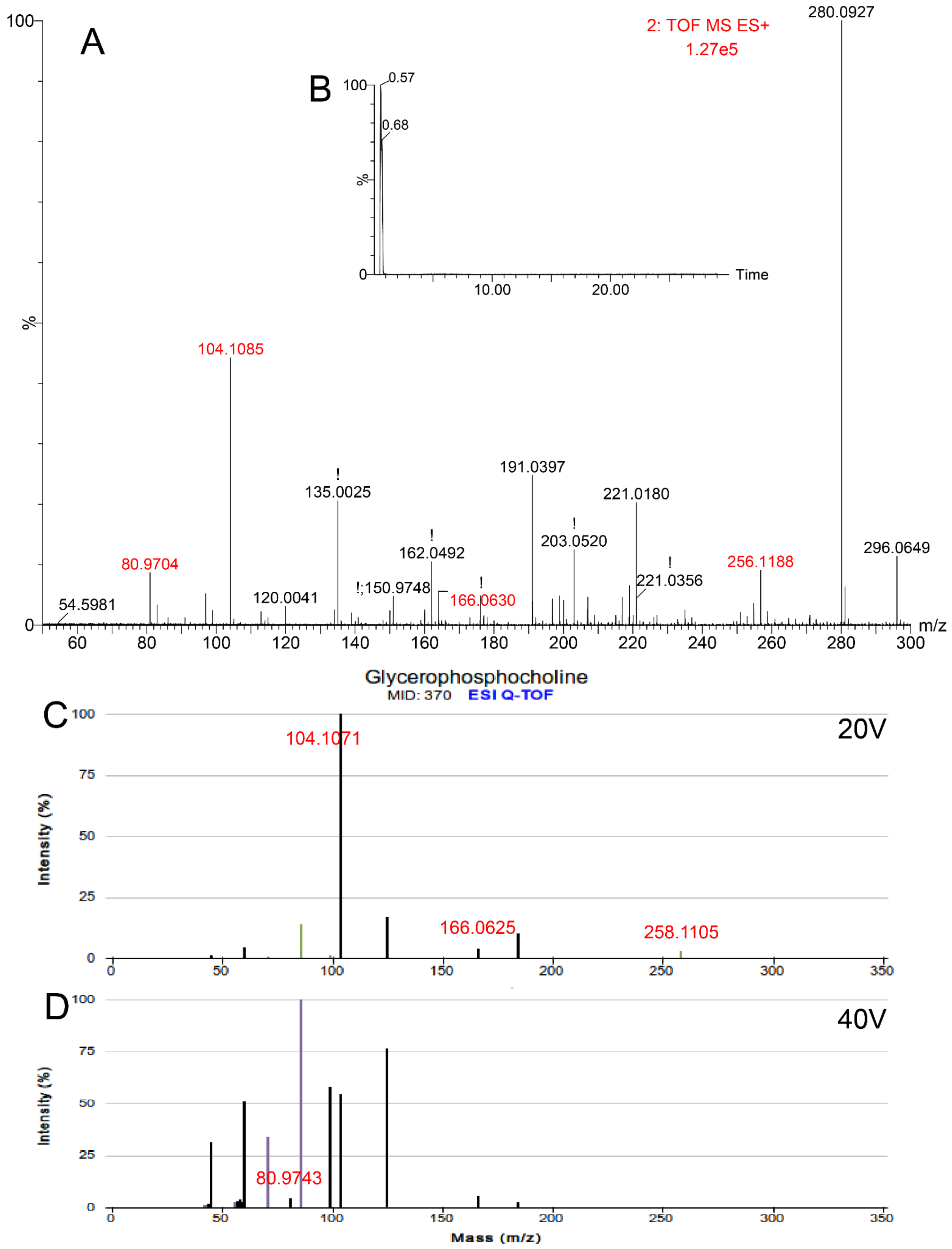

Figure S1 MS/MS spectra of the identified potential marker Glycerophosphocholine (A) and the chromatographic peak of Glycerophosphocholine (B). The reference spectra from METLIN database with different voltage 20V (C) and 40V (D).

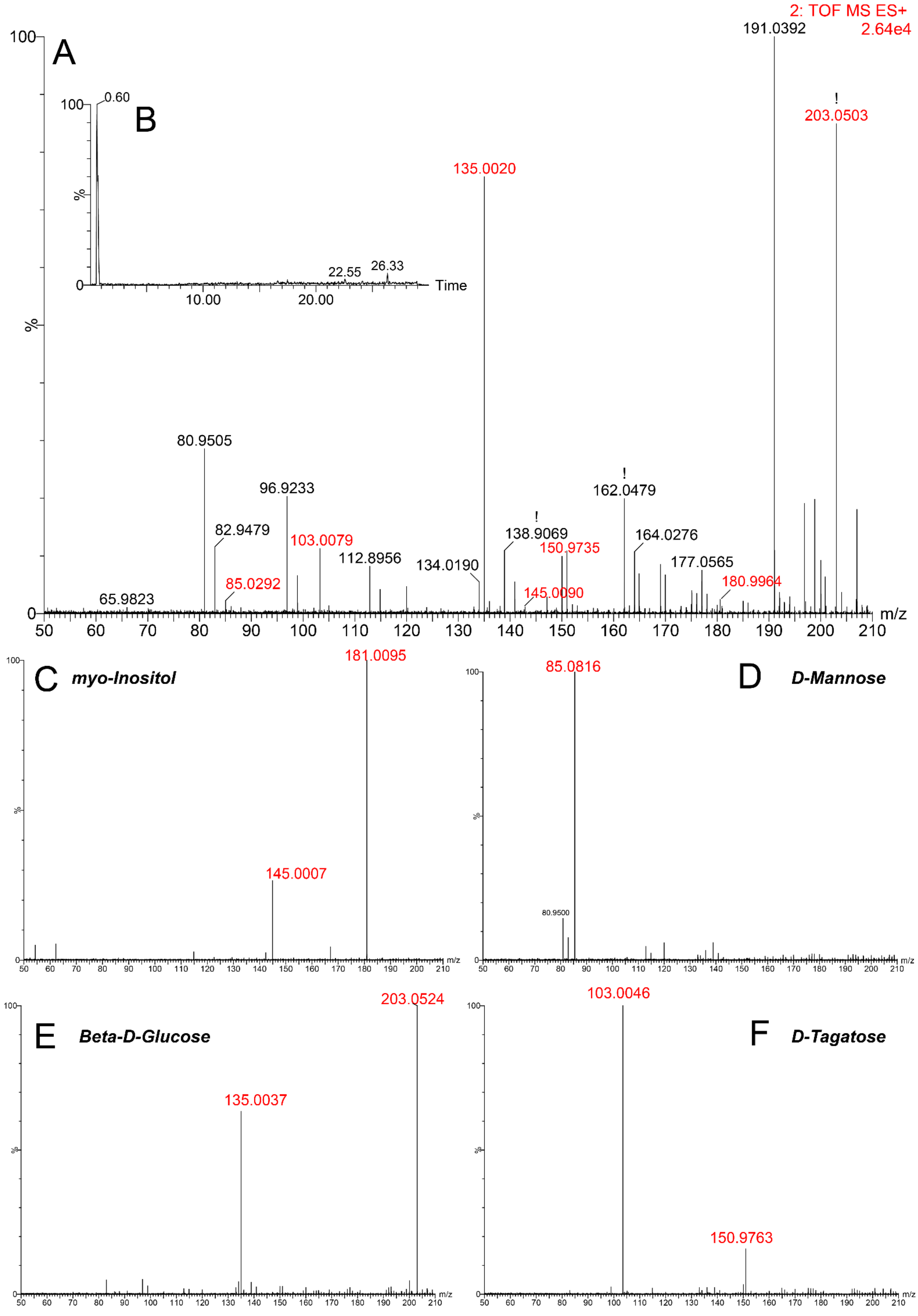
2: TOF MS ES+
2.64e4
A
B
0.60
22.55
26.33
Time
10.00
20.00
191.0392
203.0503
135.0020
80.9505
96.9233
82.9479
103.0079
112.8956
85.0292
65.9823
134.0190
138.9069
150.9735
145.0090
162.0479
164.0276
177.0565
180.9964
m/z
C myo-Inositol
181.0095
145.0007
D D-Mannose
85.0816
80.9500
E Beta-D-Glucose
203.0524
135.0037
F D-Tagatose
103.0046
150.9763

Figure S2 MS/MS spectra of the identified potential marker Monosaccharides (A) and the chromatographic peak of Glycerophosphocholine (B). The reference standard chemical spectra including Myoinositol (C), D-mannose (D), Beta-d-glucose (E) and D-tagatose (F) in experimental condition.

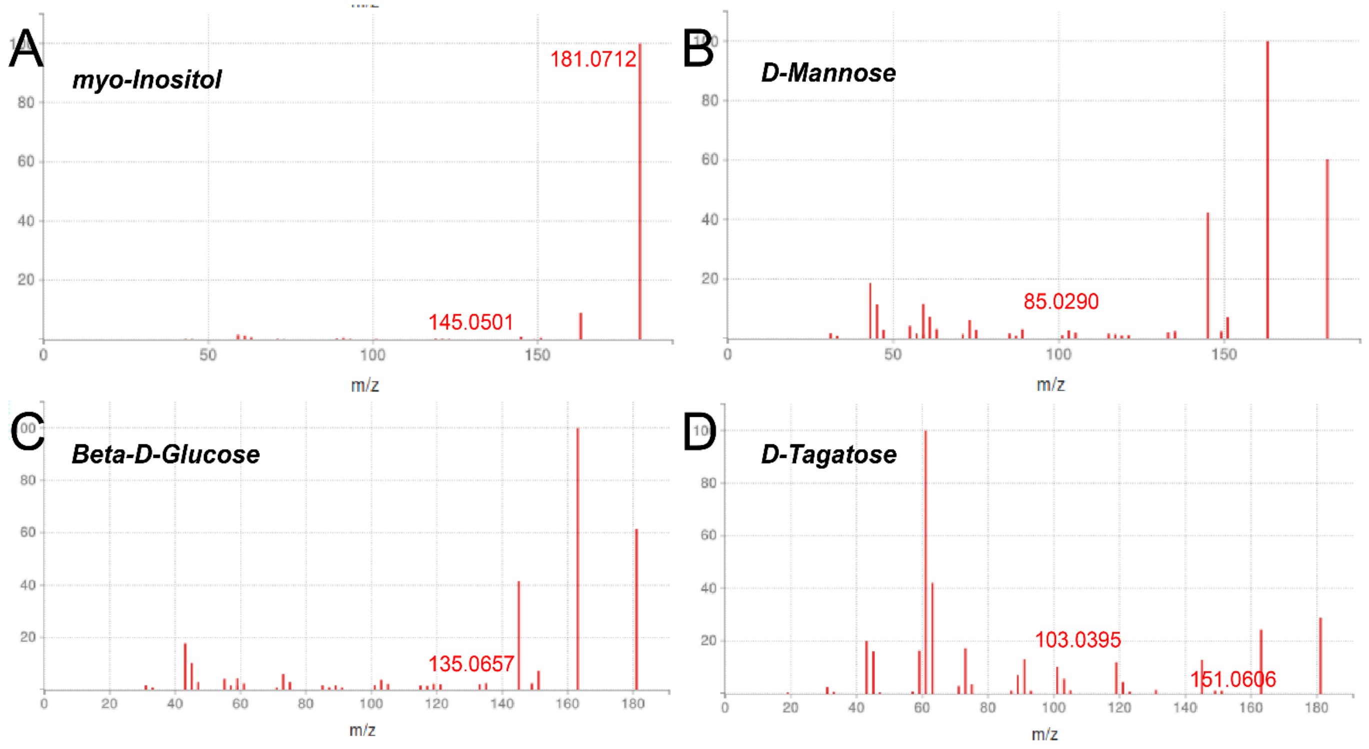

Figure S2-2 MS/MS spectra of the reference spectra from HMDB database including Myoinositol (A), D-mannose (B), Beta-d-glucose (C) and D-tagatose (D).

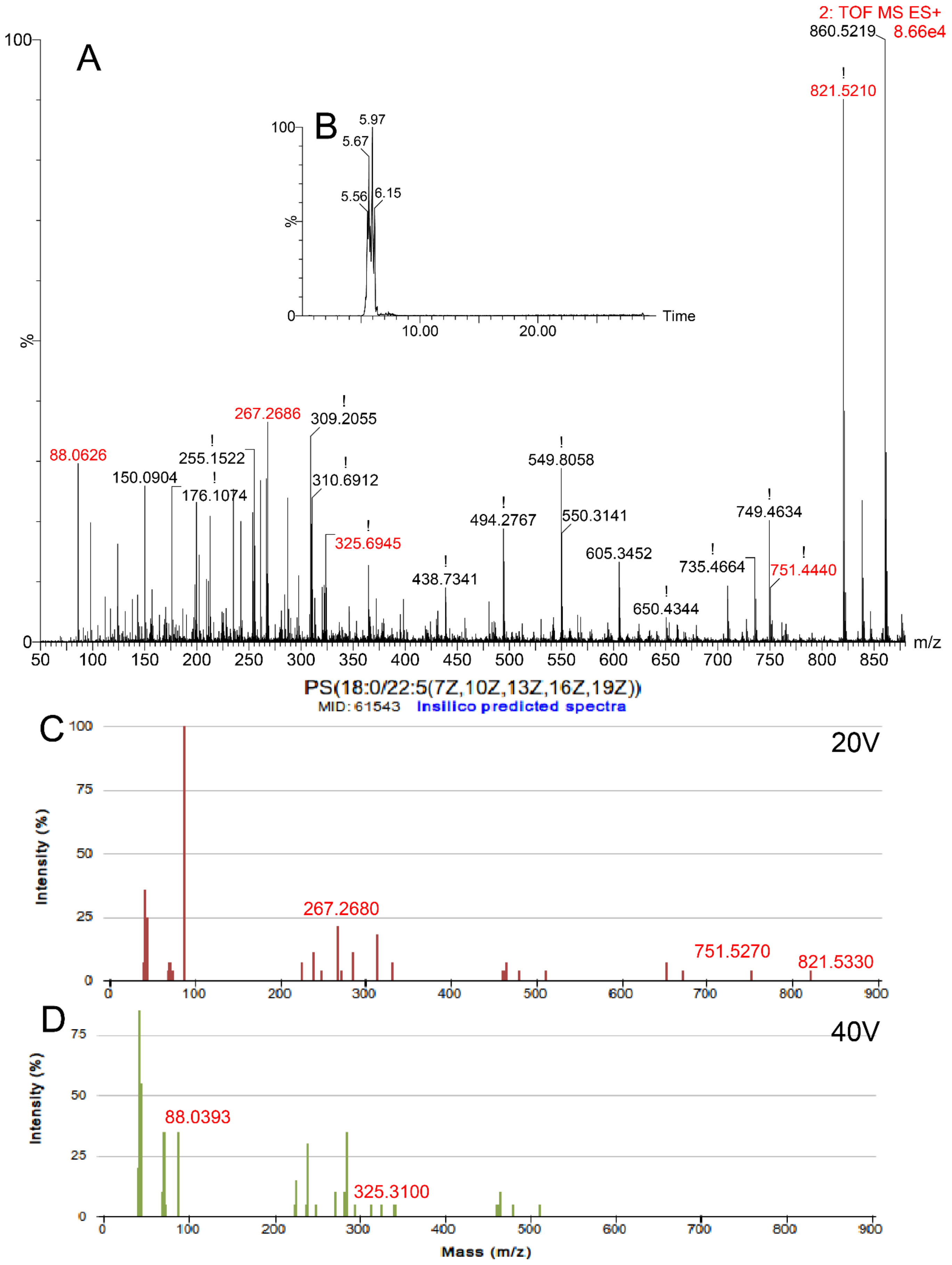

Figure S3 MS/MS spectra of the identified potential marker PS(18:0/22:5) (A) and the chromatographic peak of PS(18:0/22:5) (B). The reference spectra from METLIN database with different voltage 20V (C) and 40V (D).

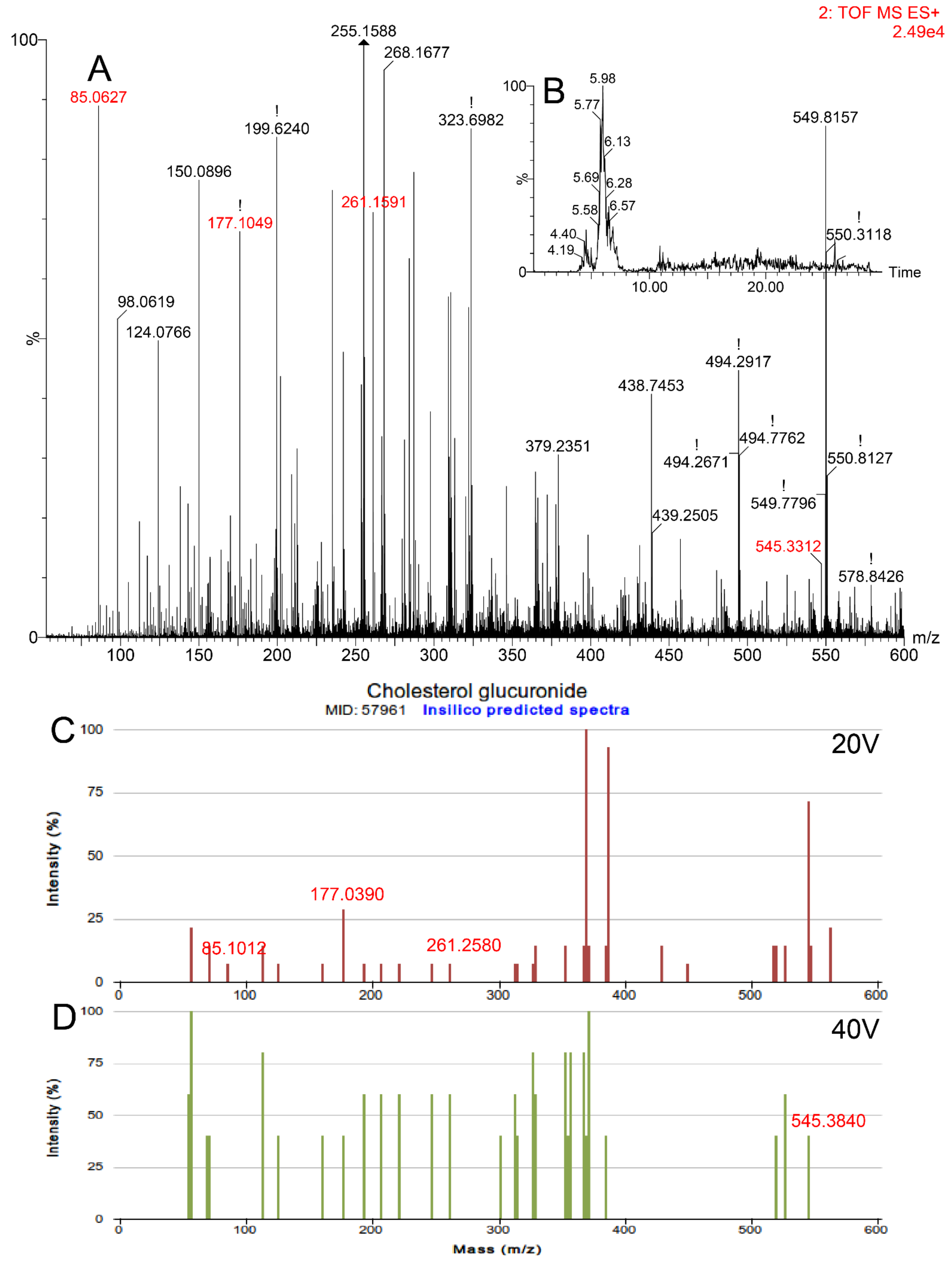

Figure S4 MS/MS spectra of the identified potential marker Cholesterol glucuronide (A) and the chromatographic peak of Cholesterol glucuronide (B). The reference spectra from METLIN database with different voltage 20V (C) and 40V (D).

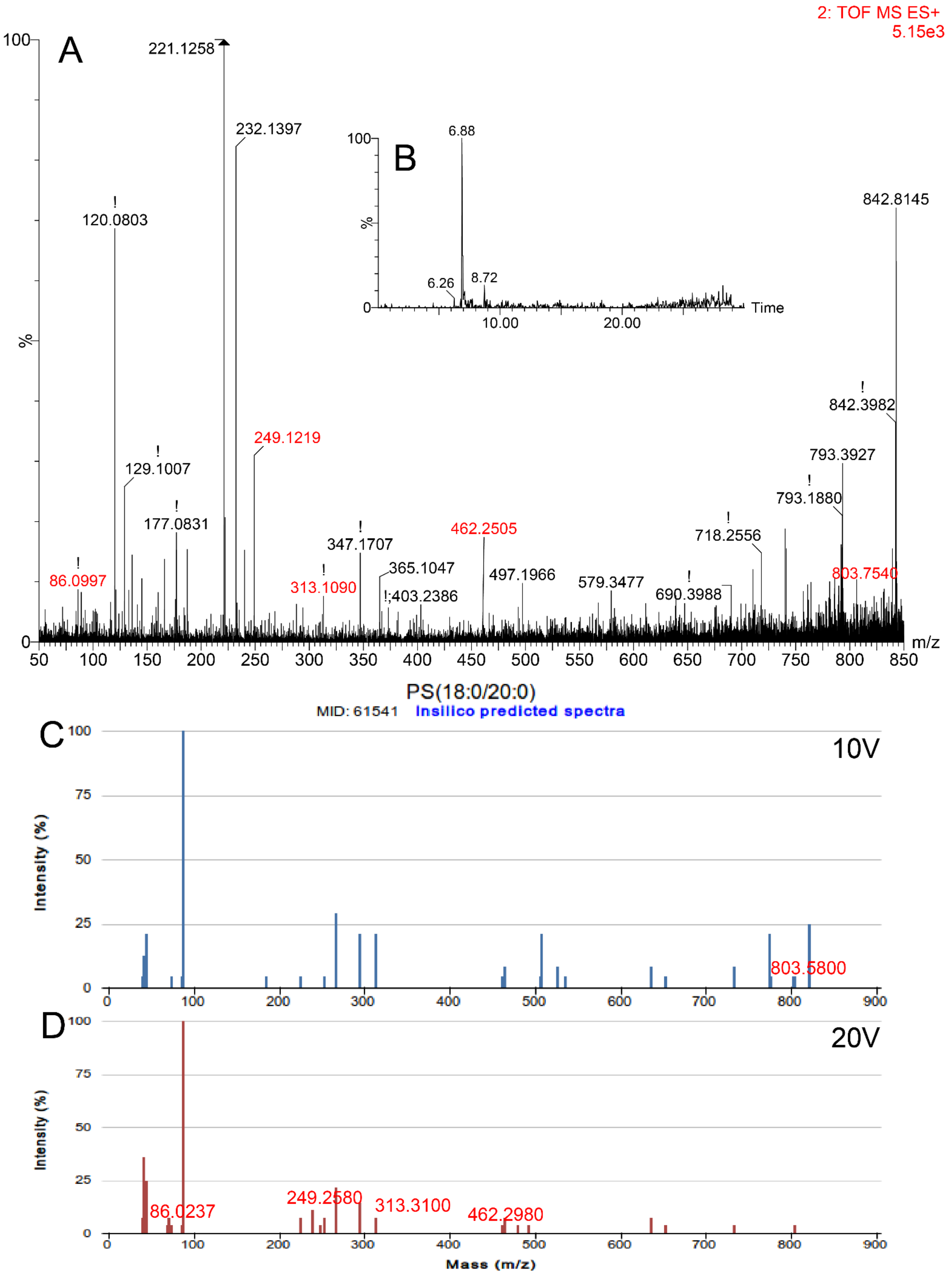

Figure S5 MS/MS spectra of the identified potential marker PS(18:0/20:0) (A) and the chromatographic peak of PS(18:0/20:0) (B). The reference spectra from METLIN database with different voltage 20V (C) and 40V (D).

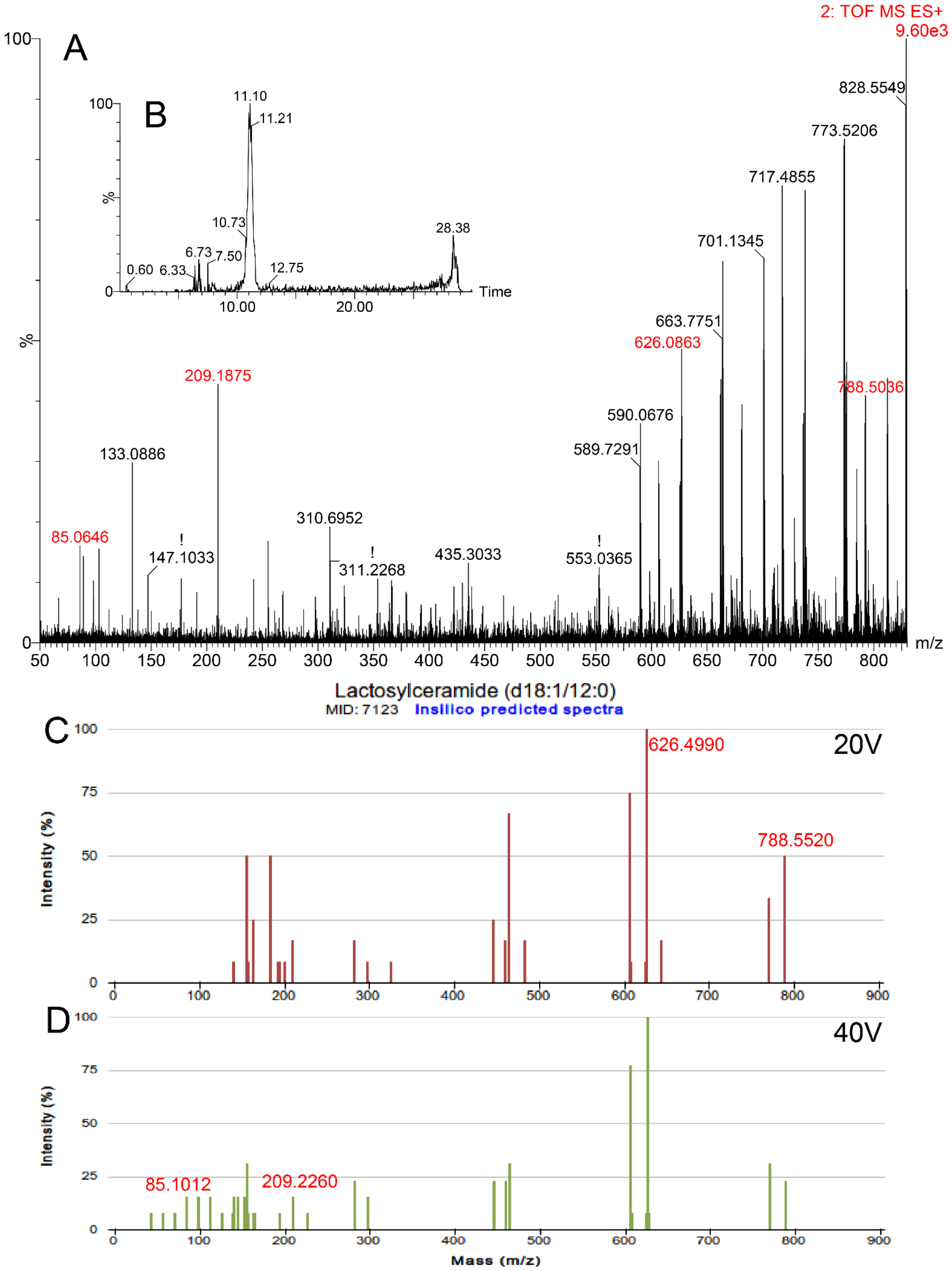

Figure S6 MS/MS spectra of the identified potential marker Lactosylceramide (d18:1/12:0) (A) and the chromatographic peak of Lactosylceramide (d18:1/12:0) (B). The reference spectra from METLIN database with different voltage 20V (C) and 40V (D).

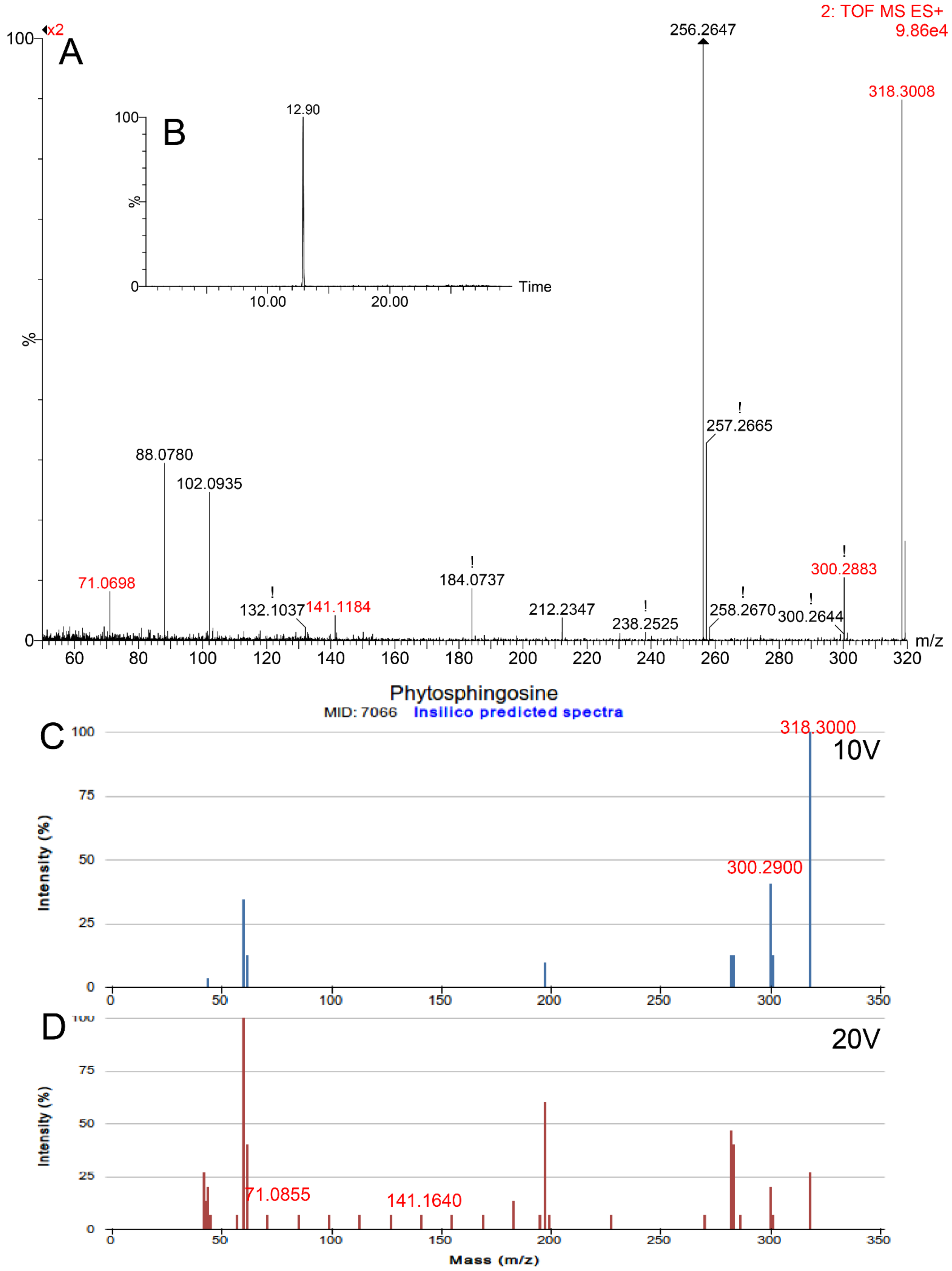

Figure S7 MS/MS spectra of the identified potential marker Phytosphingosine (A) and the chromatographic peak of Phytosphingosine (B). The reference spectra from METLIN database with different voltage 10V (C) and 20V (D).

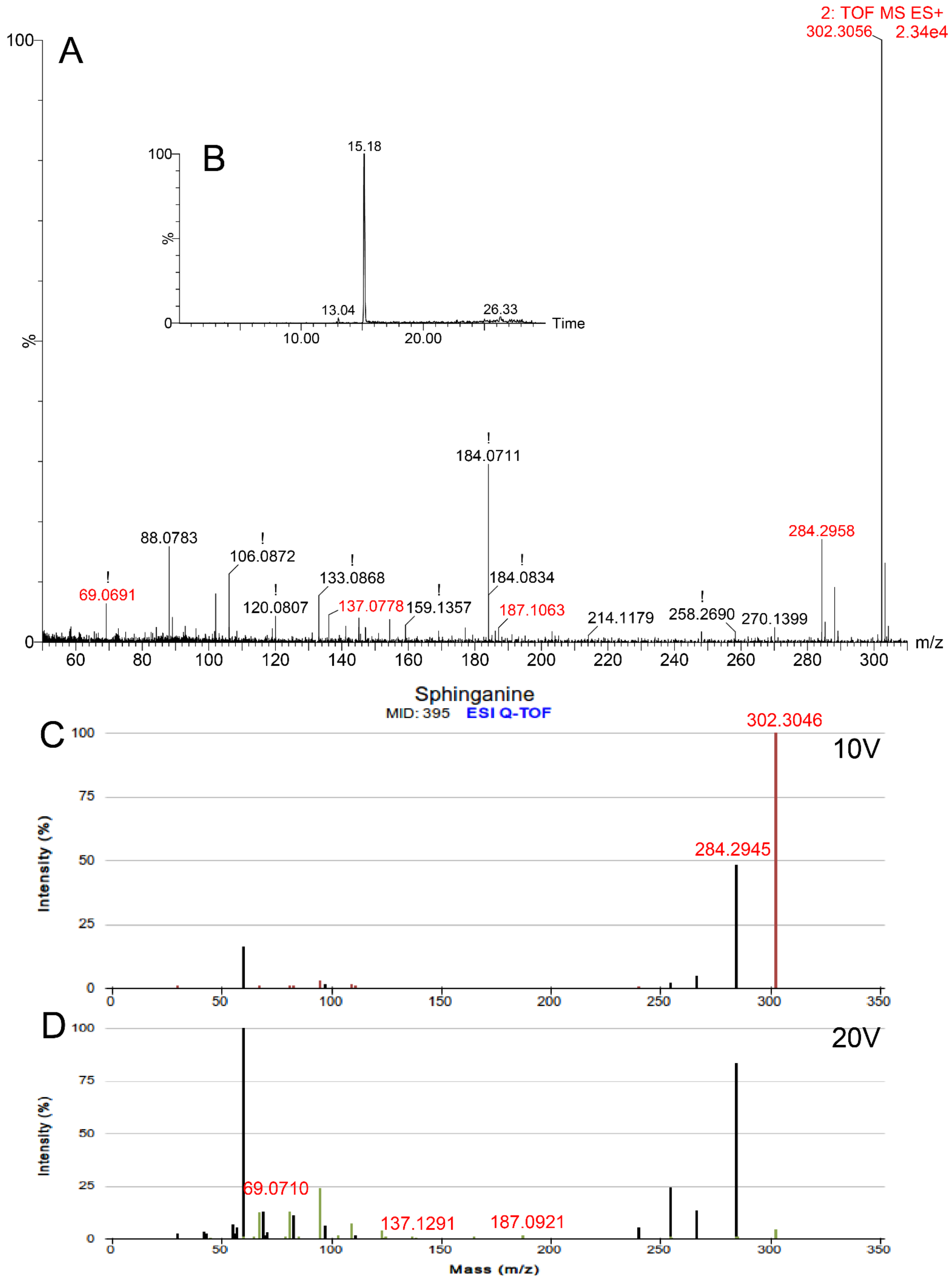

Figure S8 MS/MS spectra of the identified potential marker Sphinganine (A) and the chromatographic peak of Sphinganine (B). The reference spectra from METLIN database with different voltage 10V (C) and 20V (D).

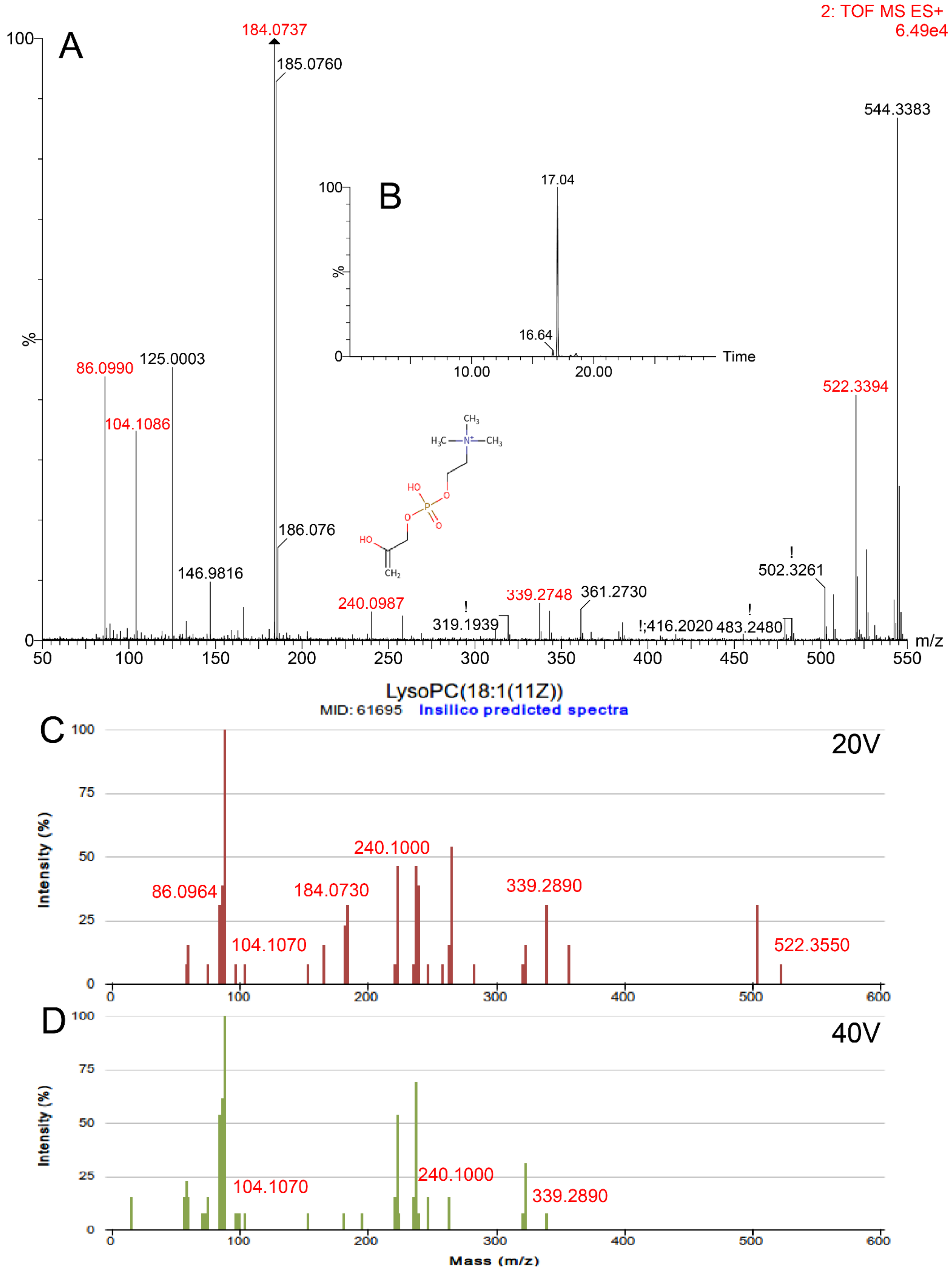

Figure S9 MS/MS spectra of the identified potential marker LysoPC (18:1) (A) and the chromatographic peak of LysoPC(18:1) (B). The reference spectra from METLIN database with different voltage 20V (C) and 40V (D).

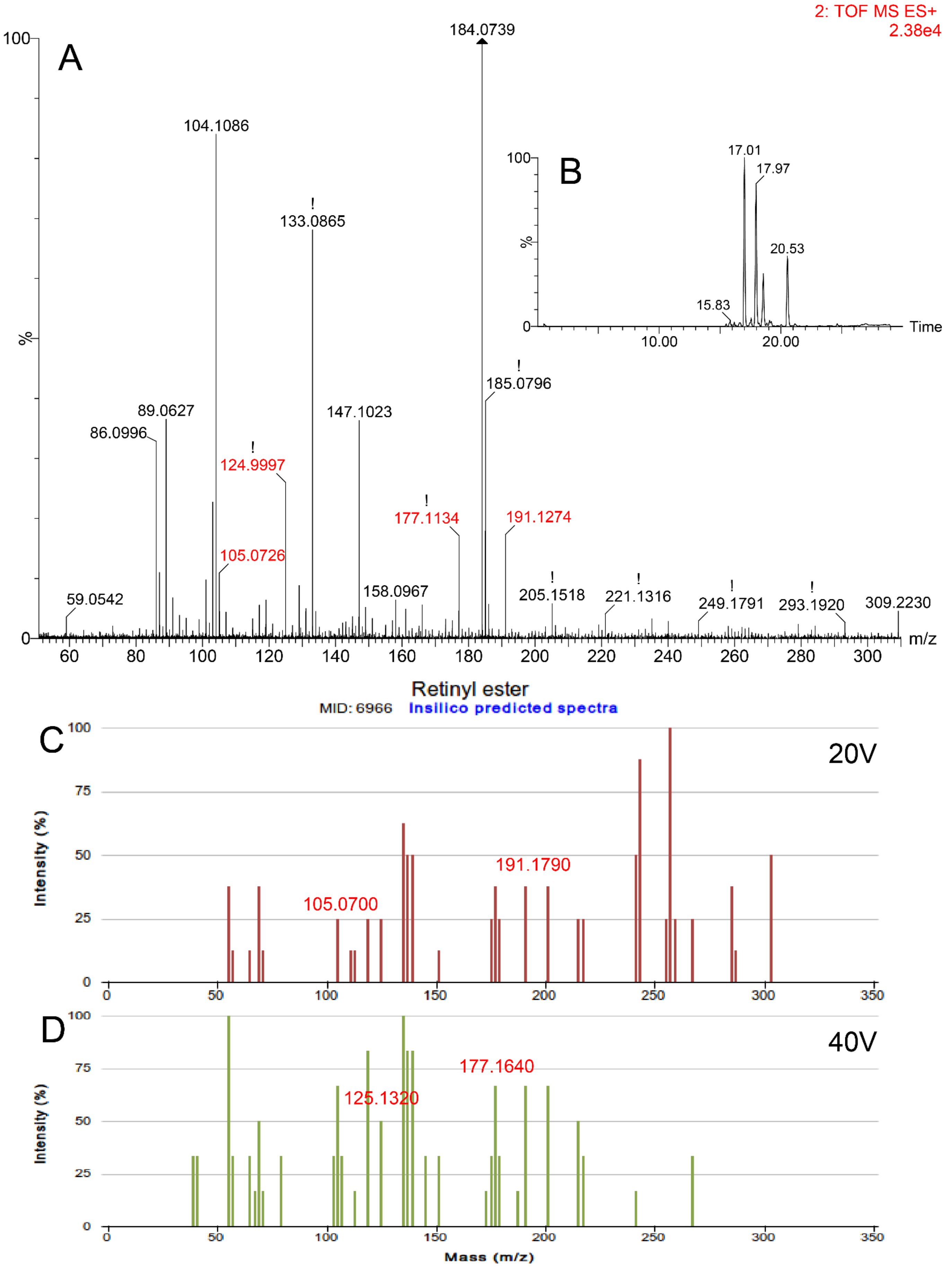

Figure S10 MS/MS spectra of the identified potential marker Retinyl ester (A) and the chromatographic peak of Retinyl ester (B). The reference spectra from METLIN database with different voltage 20V (C) and 40V (D).

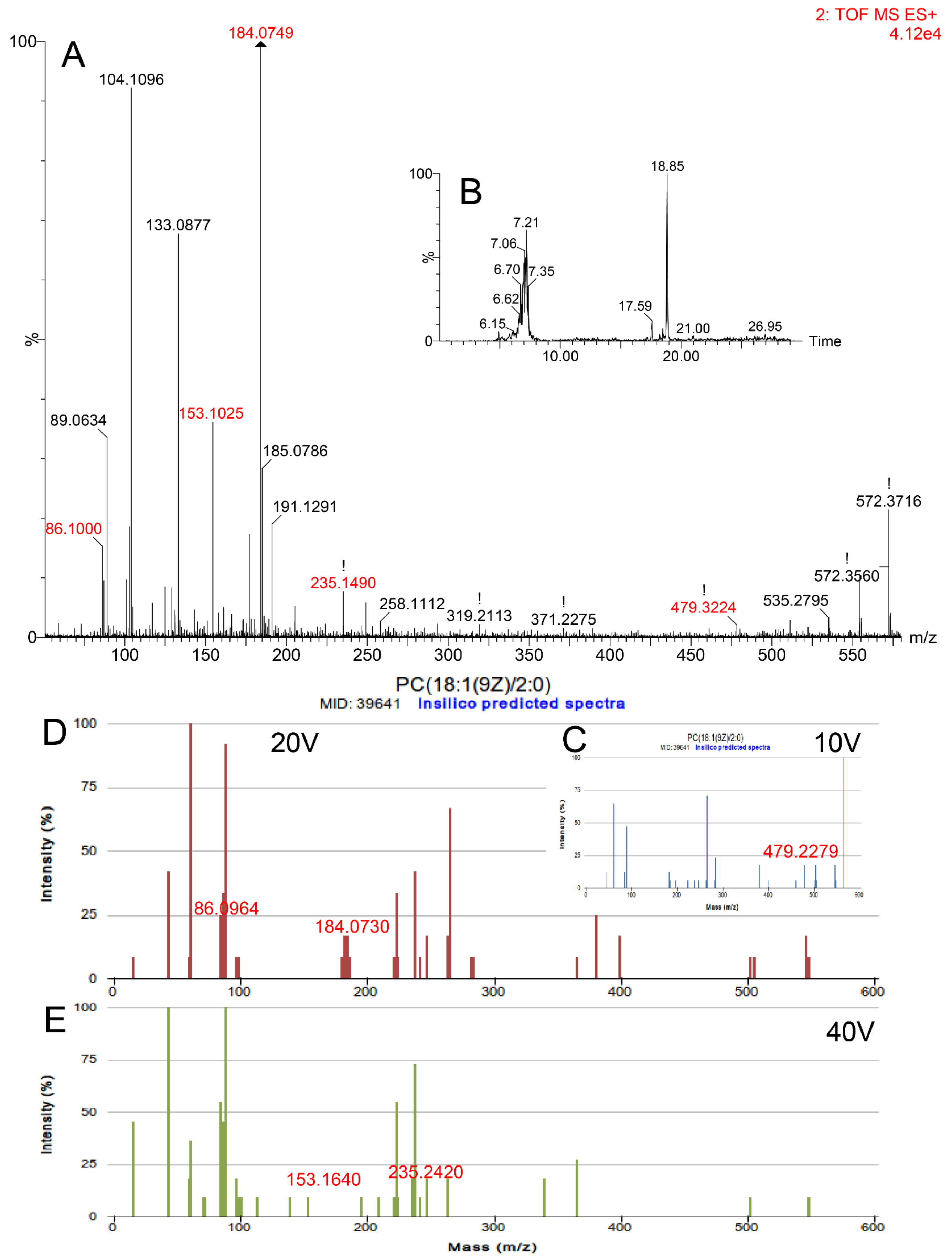

Figure S11 MS/MS spectra of the identified potential marker PC(18:1/2:0) (A) and the chromatographic peak of PC(18:1/2:0) (B). The reference spectra from METLIN database with different voltage 20V (C) and 40V (D).

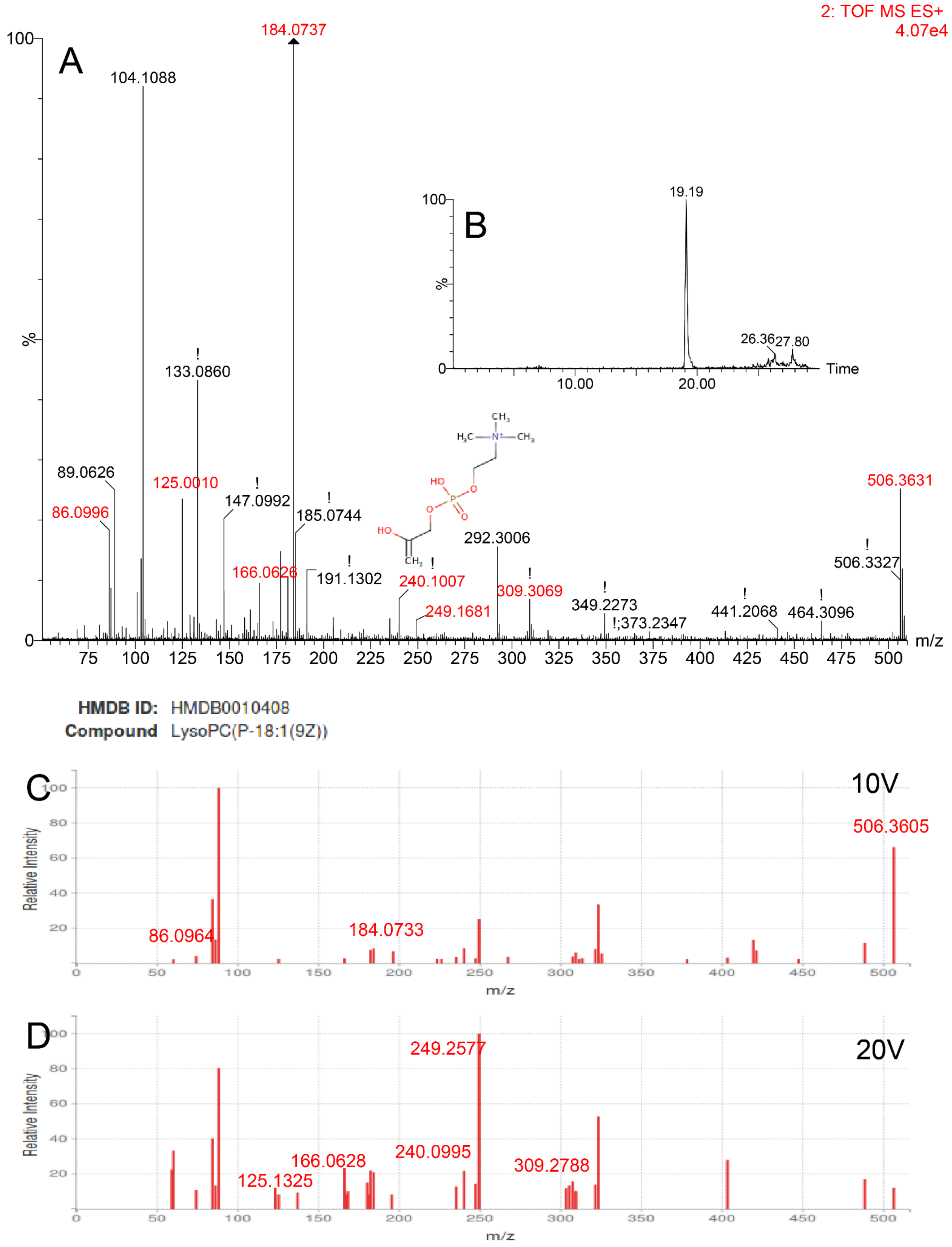

Figure S12 MS/MS spectra of the identified potential marker LysoPC(p-18:1) (A) and the chromatographic peak of LysoPC(p-18:1) (B). The reference spectra from HMDB database with different voltage 10V (C) and 20V (D).

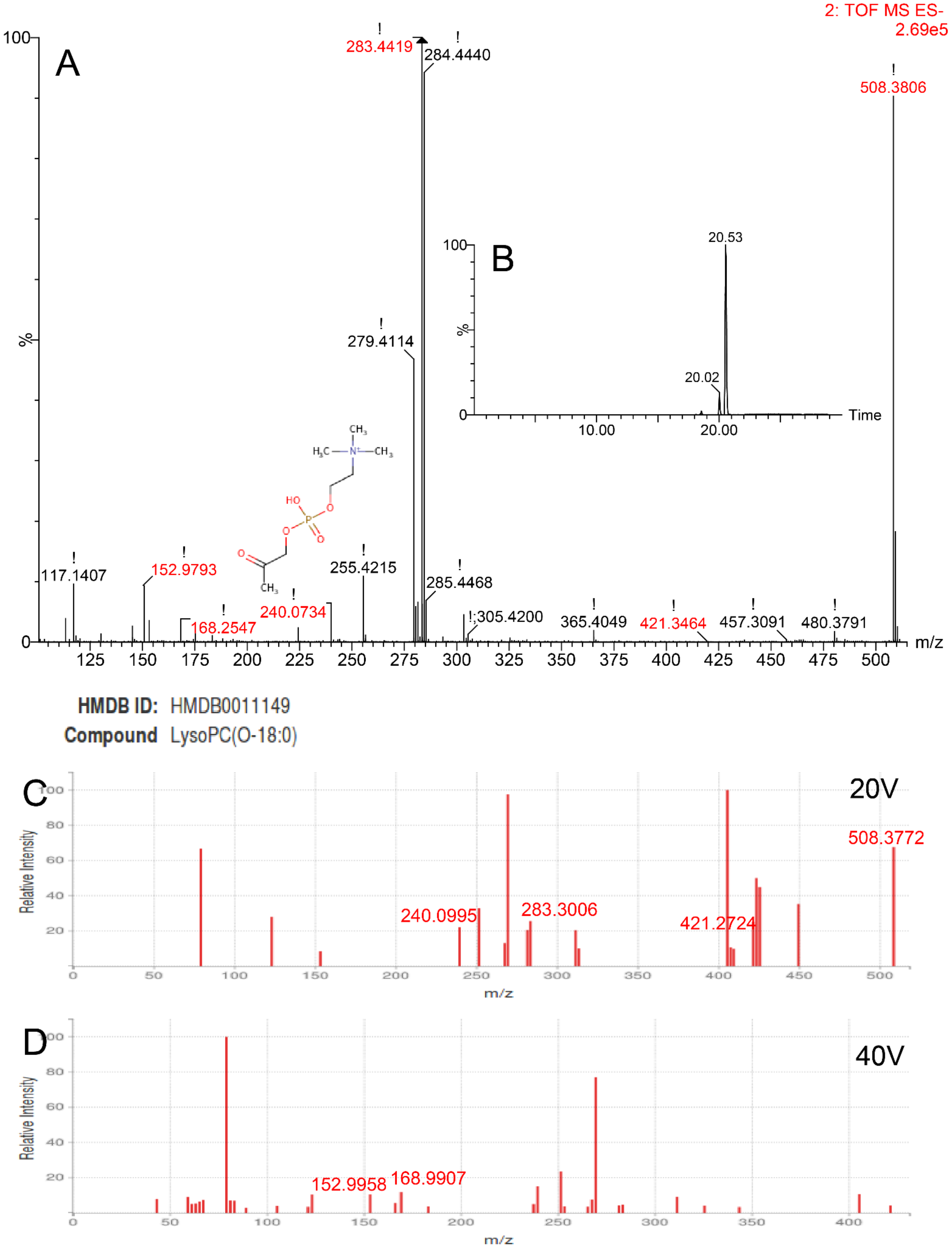

Figure S13 MS/MS spectra of the identified potential marker LysoPC(o-18:0) (A) and the chromatographic peak of LysoPC(o-18:0) (B). The reference spectra from HMDB database with different voltage 20V (C) and 40V (D).

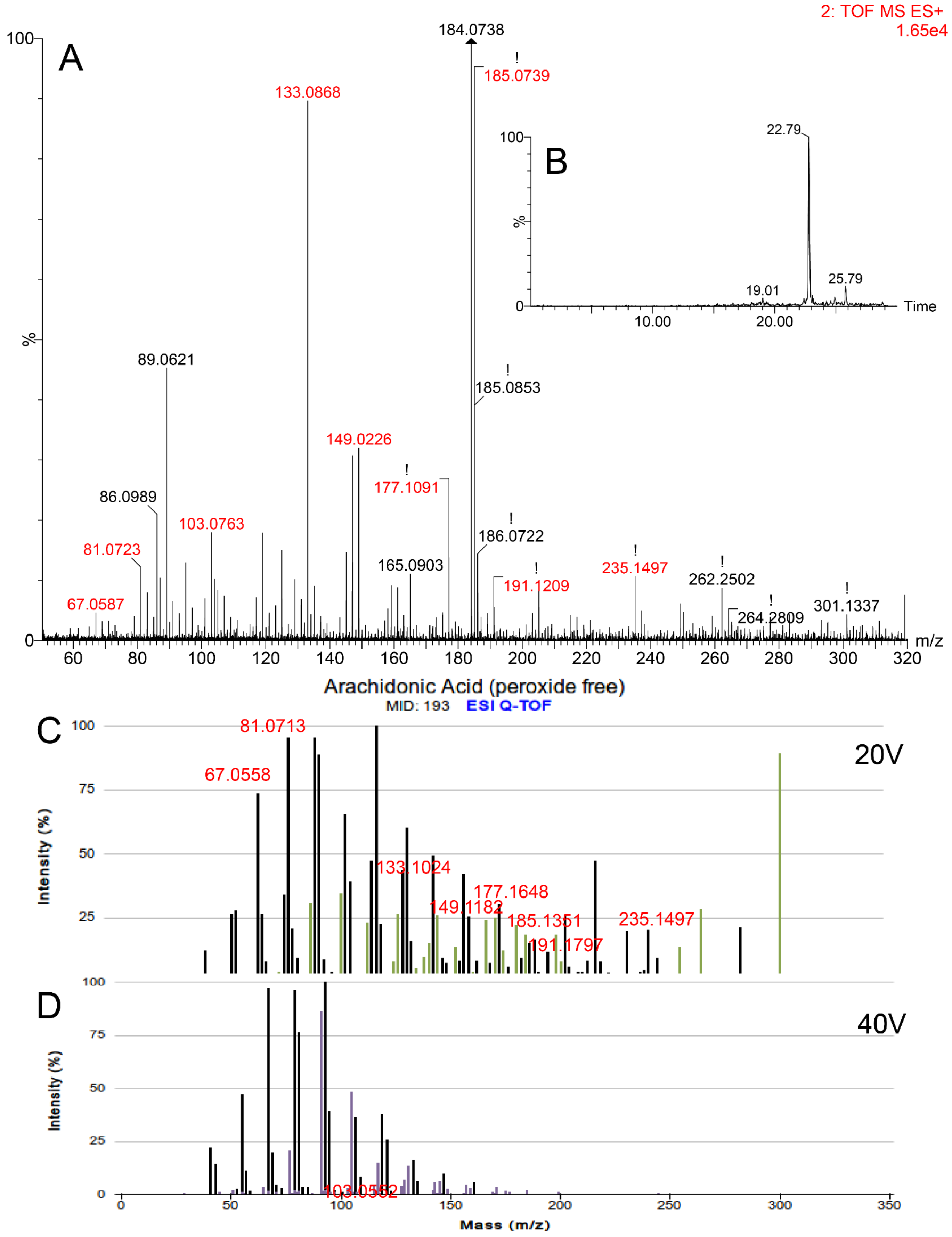

Figure S14 MS/MS spectra of the identified potential marker Arachidonic acid (A) and the chromatographic peak of Arachidonic acid (B). The reference spectra from METLIN database with different voltage 20V (C) and 40V (D).

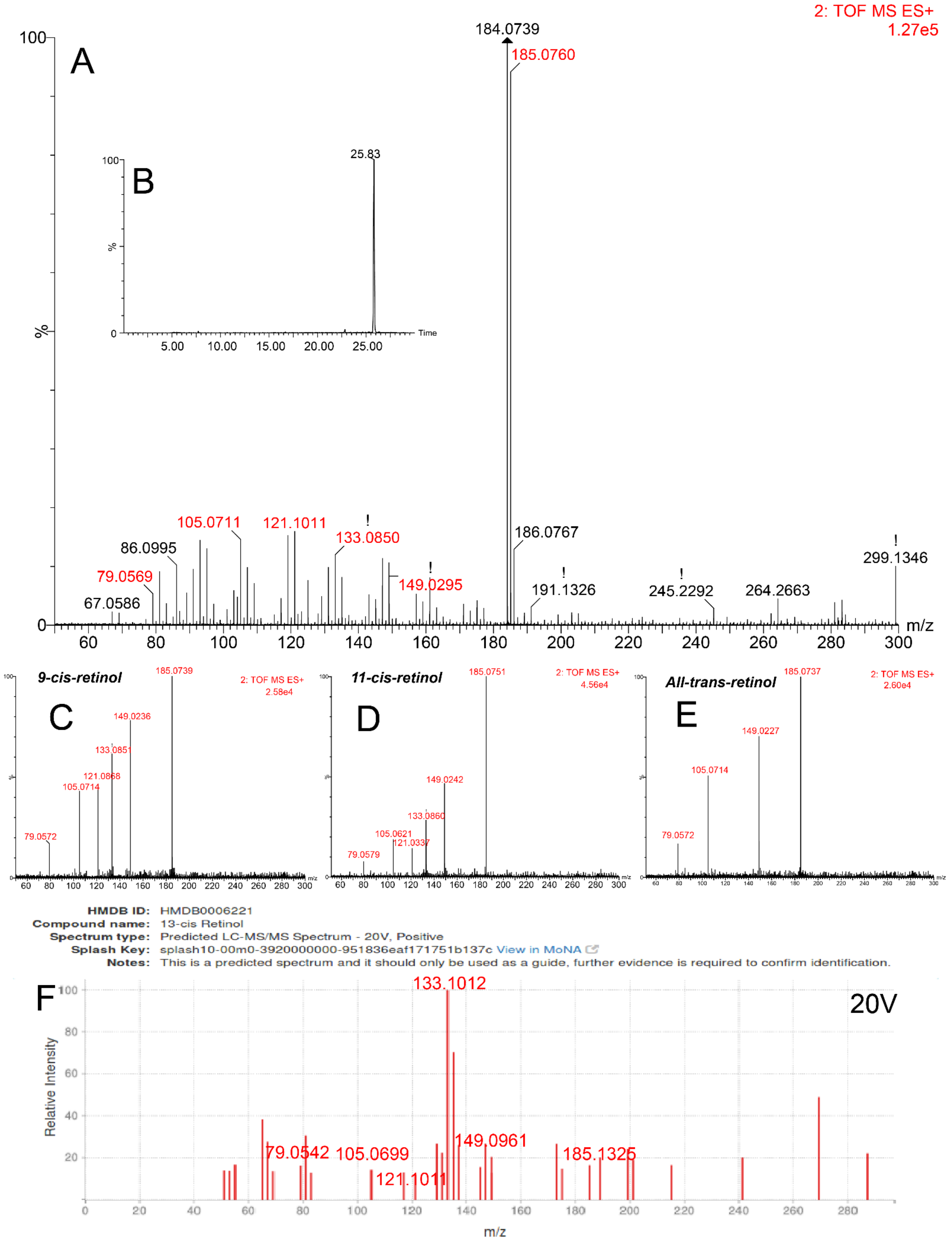

Figure S15 MS/MS spectra of the identified potential marker Retinols (A) and the chromatographic peak of Retinols (B). The reference standard chemical spectra including 9-cis-retinol (C), 11-cis-retinol (D) and all-trans-retinol (E) in experimental condition. The reference spectra from HMDB database with different voltage 20V (F).

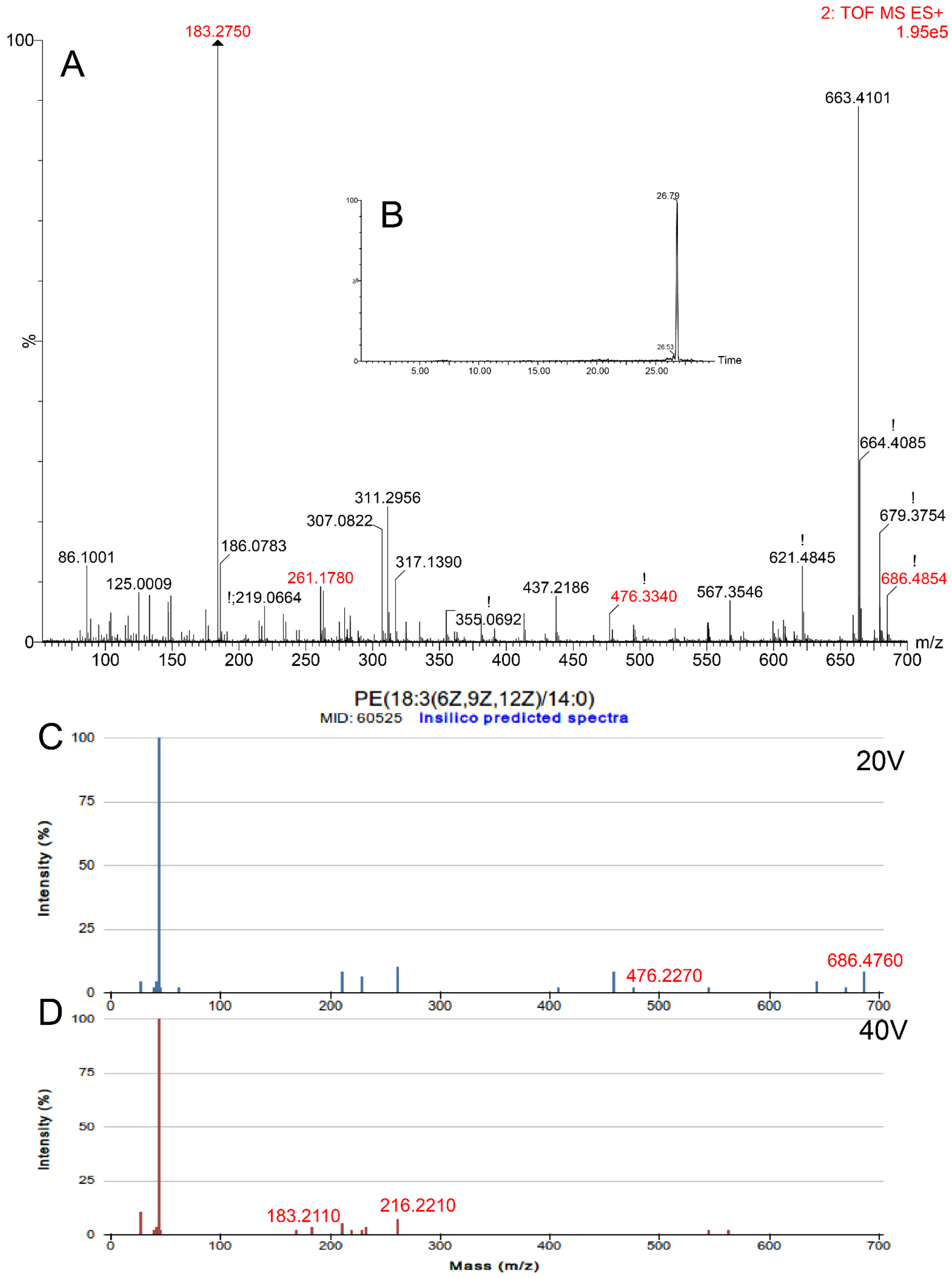

Figure S16 MS/MS spectra of the identified potential marker PE(18:3/14:0) (A) and the chromatographic peak of PE(18:3/14:0) (B). The reference spectra from METLIN database with different voltage 20V (C) and 40V (D).

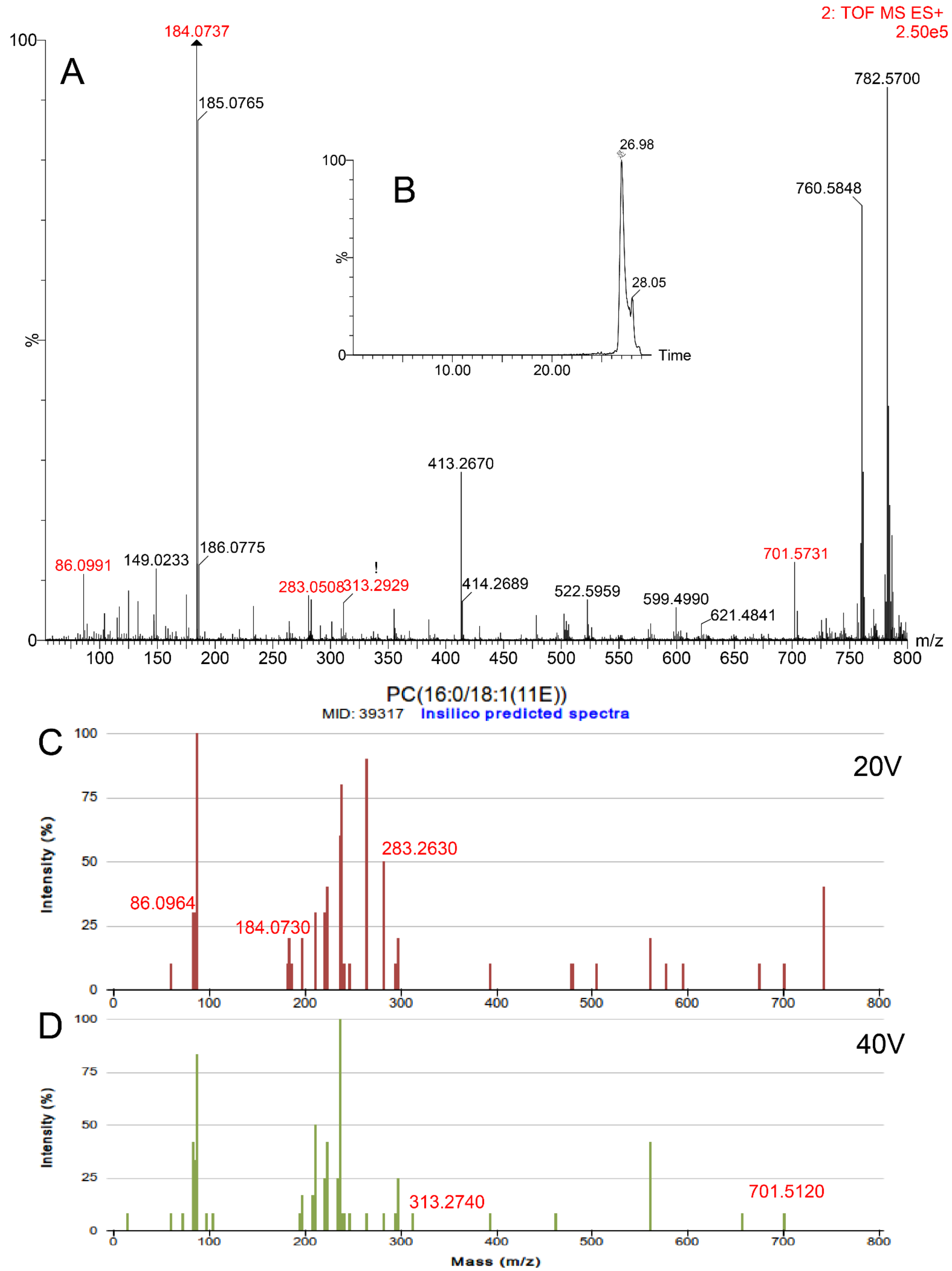

Figure S17 MS/MS spectra of the identified potential marker PC(16:0/18:1) (A) and the chromatographic peak of PC(16:0/18:1) (B). The reference spectra from METLIN database with different voltage 20V (C) and 40V (D).

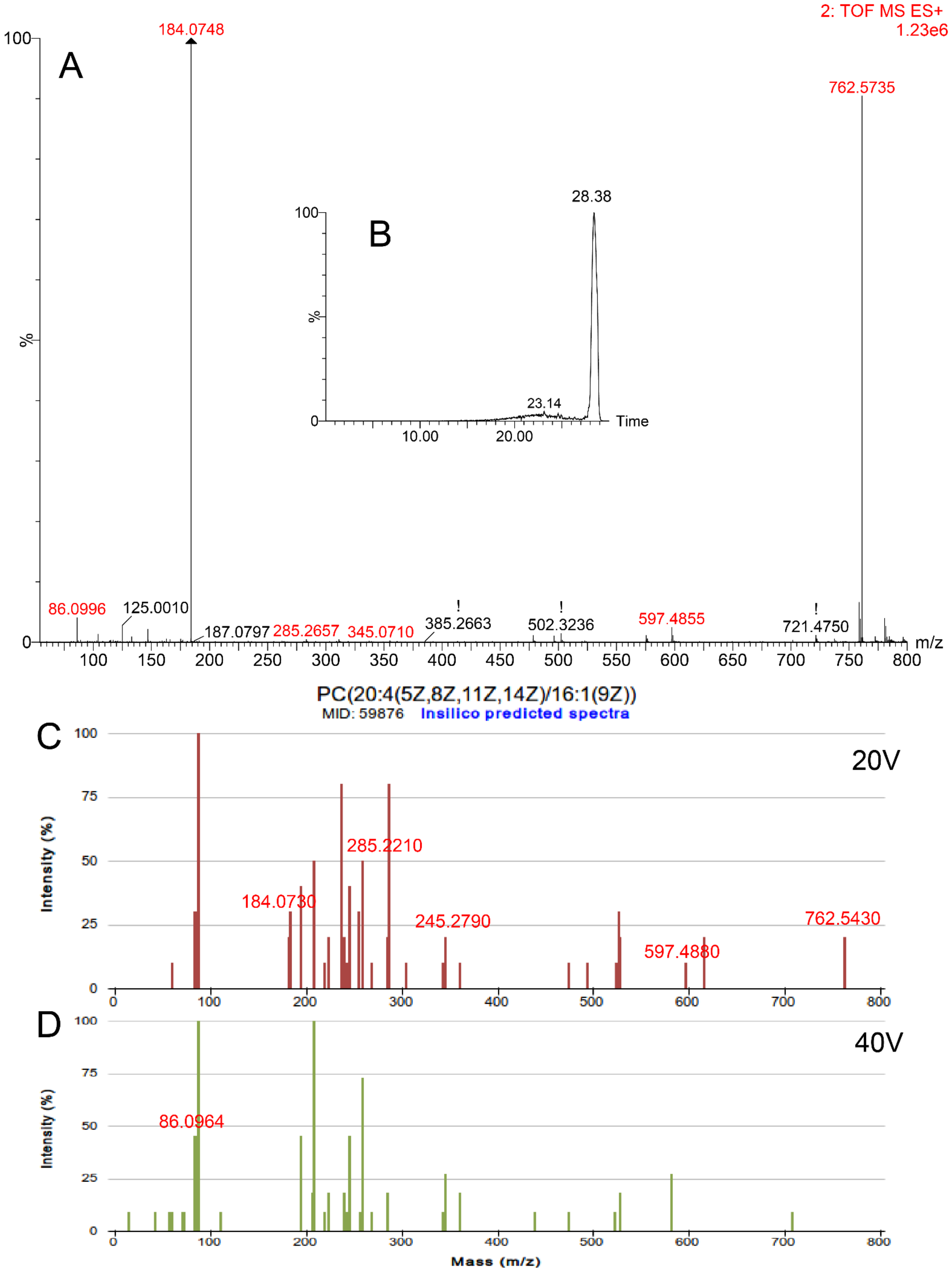

Figure S18 MS/MS spectra of the identified potential marker PC(20:4/16:1) (A) and the chromatographic peak of PC(20:4/16:1) (B). The reference spectra from METLIN database with different voltage 20V (C) and 40V (D).

Table S3 Validation results of all OPLS-DA models with leave-1/7-out approach

OPLS-DA Models	R2X	R2Y	Q2
E vs. N in ESI^+	0.463	0.792	0.595
E vs. H in ESI^+	0.37	0.938	0.744
N vs. H in ESI^+	0.403	0.773	0.557
E&N vs. H in ESI^+	0.309	0.77	0.617
E vs. N in ESI^-	0.227	0.773	-0.141
E vs. H in ESI^-	0.445	0.975	0.803
N vs. H in ESI^-	0.226	0.903	0.703
E&N vs. H in ESI^-	0.441	0.969	0.748

Table S4 The Covariation values and Correction values of all metabolites in different OPLS-DA models

OPLS-DA Model	Distinct Ion pairs	Covariation Values	Correction Values	VIP Values	P Values
ESI^+ E vs. N	12.90_318.3010	0.118397	0.755651	7.3805	7.05E-05
	0.61_280.0917	0.0365028	0.667743	2.51005	0.000326
	15.19_302.3055	0.0425538	0.474658	1.9708	0.047694643
	0.70_203.0524	0.0509496	0.457688	2.82332	0.042260598
	6.89_842.6057	0.0180737	0.459206	1.990049	0.032812373
	25.80_287.2364	0.0218074	0.370499	1.54737	0.026802839
	27.99_782.5671	-0.0574966	-0.240729	1.77642	0.030861612
	26.79_686.4818	-0.0431691	-0.418987	2.62265	0.004919327
	11.06_828.5487	-0.0130426	-0.278189	1.19411	0.031048706
	19.18_506.3594	0.0231389	0.451784	1.74855	0.005030134
ESI^+ E vs. H	12.90_318.3010	0.110799	0.769561	7.22546	2.57E-06
	0.70_203.0524	0.0466098	0.492671	3.14518	0.003468574
	28.41_780.5483	0.0524261	0.403466	3.3435	0.021292274
	15.19_302.3055	0.0379276	0.562261	2.47153	0.001230995
	0.61_280.0917	0.0348332	0.518727	2.24336	0.002970758
	5.91_860.5206	0.0325903	0.420746	1.76505	0.037576389
	25.80_287.2364	0.022717	0.398422	1.44544	0.019866047
	19.18_506.3594	0.0194385	0.376527	1.07849	0.046083784
	11.06_828.5487	-0.0103651	-0.27406	1.196258	0.0370186502
	26.79_686.4818	-0.0347006	-0.430307	2.305	0.020528406
	18.13_303.2326	-0.0231564	-0.584723	1.48192	0.003613075
ESI^+ N vs. H	12.90_318.3010	0.0790348	0.523865	4.19433	0.017677456
	0.70_203.0524	0.0407588	0.439684	2.10069	0.045296037
	5.91_860.5206	0.0214799	0.234317	2.1022	0.043300929
	28.41_780.5483	0.0143576	0.0993949	2.29905	0.012033582
	19.18_506.3594	0.00706233	0.120437	1.247319	0.03872105
	26.79_686.4818	-0.0151133	-0.267019	1.06049	0.042588939
	22.81_305.2452	-0.0162656	-0.470859	1.18809	0.003826242
	18.13_303.2326	-0.0268681	-0.539498	1.85449	0.002459153
ESI^+ E&N vs. H	12.90_318.3010	0.115972	0.730819	6.31695	7.82E-05
	0.70_203.0524	0.054102	0.525604	2.85482	0.005944511
	15.19_302.3055	0.0414287	0.507473	2.01367	0.013255539
	5.99_585.3700	0.0228178	0.493404	1.73643	0.002806233
	0.61_280.0917	0.036278	0.511807	2.23269	0.016244953
	5.91_860.5206	0.0229462	0.252635	2.07668	0.020295391
	16.65_544.3407	-0.0471518	-0.247716	3.04717	0.048562618
	27.99_782.5671	-0.0653799	-0.332702	3.39075	0.018890372
	26.79_686.4818	-0.0338479	-0.391856	1.75472	0.010120433
	11.06_828.5487	-0.00943672	-0.222903	1.340619	0.027514478
ESI^- E&N vs. H	20.06_508.3861	-0.0805057	-0.513899	3.19995	0.000395723
